# The Vesicle Protein SAM-4 Regulates the Processivity of Synaptic Vesicle Transport

**DOI:** 10.1371/journal.pgen.1004644

**Published:** 2014-10-16

**Authors:** Qun Zheng, Shikha Ahlawat, Anneliese Schaefer, Tim Mahoney, Sandhya P. Koushika, Michael L. Nonet

**Affiliations:** 1 Department of Anatomy and Neurobiology, Washington University Medical School, St. Louis, Missouri, United States of America; 2 National Centre for Biological Sciences, Tata Institute of Fundamental Research, Bangalore, India; 3 Department of Neurology, Washington University Medical School, St. Louis, Missouri, United States of America; 4 Huffington Center On Aging, Baylor College of Medicine, Houston, Texas, United States of America; 5 Department of Biological Sciences, Tata Institute of Fundamental Research, Colaba, Mumbai, India; University of California San Diego, United States of America

## Abstract

Axonal transport of synaptic vesicles (SVs) is a KIF1A/UNC-104 mediated process critical for synapse development and maintenance yet little is known of how SV transport is regulated. Using *C. elegans* as an *in vivo* model, we identified SAM-4 as a novel conserved vesicular component regulating SV transport. Processivity, but not velocity, of SV transport was reduced in *sam-4* mutants. s*am-4* displayed strong genetic interactions with mutations in the cargo binding but not the motor domain of *unc-104*. Gain-of-function mutations in the *unc-104* motor domain, identified in this study, suppress the *sam-4* defects by increasing processivity of the SV transport. Genetic analyses suggest that SAM-4, SYD-2/liprin-α and the KIF1A/UNC-104 motor function in the same pathway to regulate SV transport. Our data support a model in which the SV protein SAM-4 regulates the processivity of SV transport.

## Author Summary

Most cellular components of neurons are synthesized in the cell body and must be transported great distances to form synapses at the ends of axons and dendrites. Neurons use a specialized axonal transport system consisting of microtubule cytoskeletal tracks and numerous molecular motors to shuttle specific cargo to specific destinations in the cell. Disruption of this transport system has severe consequences to human health. Disruption of specific neuronal motors are linked to hereditary neurodegenerative conditions including forms of Charcot Marie Tooth disease, several types of hereditary spastic paraplegia, and certain forms of amyotrophic lateral sclerosis motor neuron disease. Despite recent progress in defining the cargo of many of kinesin family motors in neurons, little is known about how the activity of these transport systems is regulated. Here, using a simple invertebrate model we identify and characterize a novel protein that regulates the efficacy of the KIF1A motor that mediates transport of synaptic vesicles. These studies define a new pathway regulating SV transport with potential links to human neurological disease.

## Introduction

Neurons innervate their targets at synapses distant from the soma. Most components of these synaptic specializations, including synaptic vesicles (SVs), active zone proteins and mitochondria, are synthesized in the soma and then transported along axons on the microtubule cytoskeleton [1]. Transport along the axon is bidirectional with anterograde transport driven largely by kinesins and retrograde transport carried out by cytoplasmic dynein [2]. Efficient axonal transport is important in many facets of neuronal development and function. Trophic factors, membrane components, guidance receptors as well as synaptic components are all transported down the axon anterogradely, and maintenance of trophic support requires retrograde transport of signaling endosomes containing activated receptors [2]. Abnormal axonal trafficking has been observed in brain disorders including Parkinson's disease, amyotrophic lateral sclerosis, Charcot-Marie-Tooth disease and hereditary spastic paraplegia [3], [4], [5], [6].

The majority of anterograde transport is performed by a large family of plus-end directed motors of the kinesin superfamily (KIFs) consisting of 21 genes in *C. elegans* [7] and 45 genes in mouse [8]. KIFs are composed of three domains: a motor “head” domain, a stalk domain and a cargo-binding domain. In plus end directed kinesins, the globular ATPase motor domain is positioned in the N-terminal region of the protein and provides the force to walk processively on microtubules at mean velocities of around 0.5–1.5 µm/second [9]. The C-terminal cargo-binding domain is typically separated from the motor by a long coiled coil stalk or “neck” domain, though the size of this domain varies considerably within the family. By contrast with the highly conserved motor domain, the cargo binding domains are variable and determine the cargo specificity of KIFs. Accessory light chain subunits and distinct adaptor proteins provide additional diversity of cargo binding to KIFs [9]. For example, KIF5 binds APP containing vesicles via its light chain [10], mitochondria via the adaptor Milton [11] and GlrR2 containing vesicles via the adaptor GRIP [12]. Although the cargo binding specificity of numerous kinesins has been defined to some extent, the mechanisms regulating many aspects of kinesin-mediated cargo transport remain largely uncharacterized.

One general theme in the mechanisms controlling axonal transport is the regulation of KIF motor activity. The activity of the motor domain of several different KIFs, including KIF1A/UNC-104, is negatively regulated by their cargo-binding domain in the absence of cargo [13], [14], [15], [16], [17], [18]. In addition, activation of the motor in several cases has been documented to require the binding of other factors. For example, the cargo adaptor JIP1 is not sufficient to activate Kinesin-1, but rather requires the additional cooperative binding of the protein FEZ1 [19]. A RAN-GTPase binding protein has been shown to activate Kinesin-1 ATP activity *in vitro* [20]. Phosphorylation has also been demonstrated in several cases to regulate cargo binding. For example, CaMKII regulates KIF17 binding to cargo by phosphorylation and GSK3β phosphorylation regulates KIF5 [21], [22]. In addition, the microtubule associate protein (MAP) doublecortin was recently demonstrated to regulate SV transport by enhancing KIF1A motor domain binding to MTs [23]. In summary, regulation of KIF motor activity is complex.

One of the identified KIF1A/UNC-104 regulators is Liprin-α/SYD-2. Liprin-α/SYD-2 belongs to a family of proteins that interact with the cytosolic domain of LAR receptor protein tyrosine phosphatases [24], [25]. In addition to interacting with LAR, Liprin-α interacts with several presynaptic active zone proteins to regulate active zone development [26], [27], . Interestingly, biochemical studies also identified interactions of Liprin-α with KIF1A [32]. *In vivo*, Liprin-α/SYD-2 is required for SV trafficking in *Drosophila* [33] and regulates UNC-104 motility in *C. elegans* [34]. These observations demonstrate that, in addition to the intra-molecular regulation of KIF1A/UNC-104, its activities are also regulated by other factors.

Here, using the *C. elegans* mechanosensory system as an *in vivo* model, we identify SAM-4 (Synaptic vesicle tag Abnormal in Mechanosensory neurons) as a novel regulator of KIF1A/UNC-104 directed SV trafficking. s*am-4*, encodes a conserved SV-associated protein orthologous to human LOH12CR1 [35] that is broadly expressed in neuronal tissue. SAM-4 acts in a cell autonomous manner by binding to SVs to regulate the processivity of anterograde SV transport. *sam-4* null mutants show SV trafficking defects in different neuronal cell types. Genetic analyses revealed that SAM-4 acts synergistically with the KIF1A/UNC-104 PH cargo binding domain, but not the motor domain, to regulate SV trafficking and locomotory behavior. Gain-of-function mutations in the *unc-104* motor domain suppress *sam-4* defects indicating that SAM-4 functions upstream of the motor in regulating SV transport. SYD-2, which regulates SV trafficking to a lesser extent than SAM-4, exhibits similar genetic interactions with UNC-104 but no obvious interactions with SAM-4, consistent with SYD-2 and SAM-4 acting in the same pathway. Imaging of SV cargo movements *in vivo* demonstrated that SAM-4 is required to maintain cargo processivity rather than motor velocity, while gain-of-function UNC-104 proteins increase cargo processivity. We propose a model in which SV-bound SAM-4 acts in parallel to the UNC-104/KIF1A cargo binding domain to regulate activity of the motor domain.

## Results

### *sam-4* mutants accumulate SVs in the soma and proximal neurite

**Figure 1:**
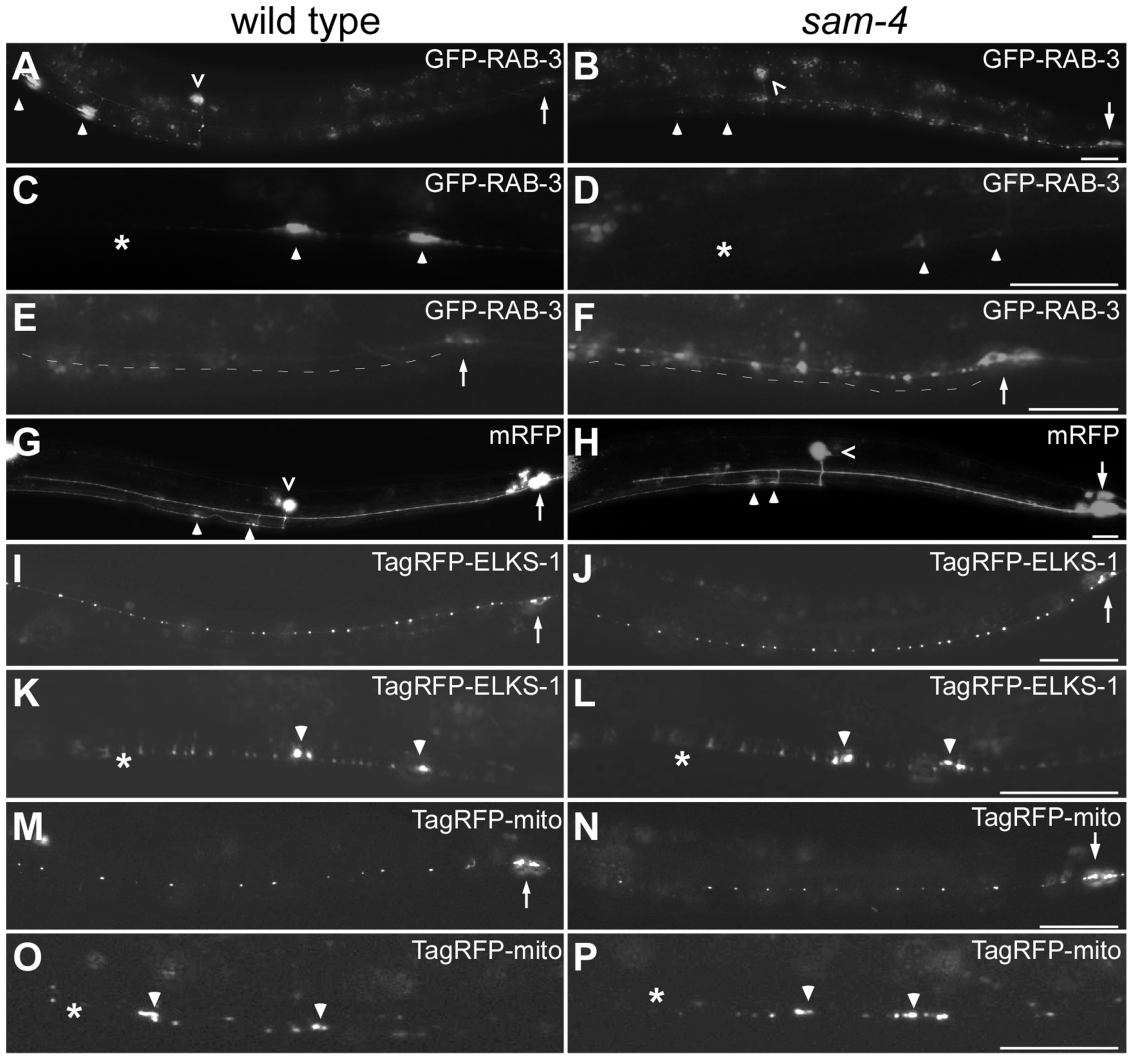
*sam-4(js415)* mutations cause abnormal accumulations of SV markers in PLM neurons. (A–H) Distribution of synaptic vesicle marker GFP-RAB-3 (*jsIs821*, A–F) accumulations in PLMs labeled by the cytosolic mRFP (*jsIs973*, G and H) in wild type animals (A, C, E, and G) and *sam-4* mutants (B, D, F, and H). Shown are PLM neurons (A, B, G and H), their synaptic varicosities (C, D) and proximal neurites (E, F). (I–L) Distribution of active zone marker TagRFP-ELKS-1 (*jsIs1075*) accumulations in PLM neurites (I, J) and synaptic varicosities (K, L). (M–P) Distribution of mitochondria (labeled by Tag-RFP-mito, *jsIs1073*) in PLM neurites (M, N) and synaptic varicosities (O, P). Arrow: PLM soma; arrowhead: PLM synaptic varicosity; asterisk: vulva; caret: PVM soma. Scale bar: 20 µm.

The response to gentle touch to the body in *C. elegans* is mediated by a set of six touch receptor neurons (TRNs: ALML/R, AVM, PLML/R, and PVM) (Figure S1A and [36]). We use PLM neurons as a simple *in vivo* system to examine axonal transport of synaptic components. The two PLM soma are located on each side of the body in the tail ganglia (Figure S1A). Each PLM extends a short posterior-directed and a long anterior-directed neurite, which are easy to image because they are in close apposition to the cuticle. PLMs innervate partners via gap junctions and chemical synapses [37]. The chemical synapses are formed in a large varicosity (∼5 µm long), located at the end of single collateral synaptic branch that extends ventrally from the anterior directed process into the ventral nerve cord, usually just posterior to the vulva (Figure S1A). We examined PLM neurons *in vivo* by expressing markers using the *mec-7* promoter which drives gene transcription selectively in TRNs [38]. SVs preferentially accumulate in the PLM synaptic varicosities as observed using transgenic SV markers SNB-1-GFP [39] and GFP-RAB-3 (*jsIs821*, Figure 1A and 1C), similar to SV accumulations revealed at the ultrastructural level [37], [40]. When anterograde SV trafficking machinery is disrupted by lesioning the UNC-104/KIF1A motor, SV markers accumulate in the soma and proximal portions of PLM neurites rather than being transported to the synapse (Figure S1B–D′). By contrast, when the retrograde cytoplasmic dynein motor is disrupted, SV markers accumulate abnormally at the distal portions of the anterior process [41]. These observations indicate that homeostatic regulation of SV levels in mechanosensory neuron synaptic varicosities is mediated by the balance of the anterograde and retrograde transport systems.

The *sam-4(js415)* mutant was isolated in a forward genetic screen for mutations disrupting SV accumulation in PLM synapses, using a SNB-1-GFP transgenic marker [42]. Similar defects were observed when SV localization was analyzed using GFP-RAB-3 (Figure 1A–F). In *sam-4(js415)*, GFP-RAB-3 fluorescence was greatly reduced in PLM synaptic varicosities (Figure 1B and D) and increased both in the soma and the process proximal to the soma where the accumulations were largely punctate (Figure 1B and F). We also found that the accumulation phenotype in PLM neurons is temperature sensitive: mutants raised at 25°C exhibit more severe defects than those raised at 15°C (Figure S2). In addition to the abnormal SV marker accumulations in PLM neurons, similar defects were also observed in other neurons including SAB neurons and ventral nerve cord neurons when using either SNB-1-GFP or GFP-RAB-3 SV markers (Figure S3A–G) [39], [43]. Thus, SAM-4 appears to be essential for efficient transport of SVs in different types of neurons.

The altered distribution of GFP-RAB3 that we observe in *sam-4* mutants could be explained by the disruption of neuronal morphology and/or the microtubule cytoskeleton. To test if the reduced levels of SV markers in *sam-4* PLM synaptic varicosities are caused by PLM anatomical defects, we examined PLM neurites using a cytosolic fluorescent marker mRFP (*jsIs973*) and found no obvious morphological changes: PLM neurites extend normally, terminate properly in the mid-body, form the synaptic branches at the appropriate location and form synaptic varicosities in the ventral nerve cord (Figure 1G and H). Since microtubules function as a common track for the anterograde transport of many synaptic components including SVs, mitochondria and active zone proteins [1], we asked if *sam-4* mutations cause microtubule cytoskeleton disruptions by examining the localization of synaptic components other than SVs. We found that the distribution of active zone proteins (*mec-7p*::tagRFP-ELKS-1, *jsIs1075*; Figure 1I–L) and mitochondria (*mec-7p*::tagRFP-mito, *jsIs1073*) (Figure 1M–P) is grossly normal in *sam-4* mutants, indicating that transport of other synaptic components is largely intact. Thus, the microtubule cytoskeleton remains competent for axonal transport.

To evaluate systemic effects of *sam-4* mutations, we next examined locomotion behavior which has been associated with SV trafficking defects [44]. Surprisingly, *sam-4* null (see below) mutants exhibit only mild defects in the velocity of stimulated locomotion and show little, if any, defects in posture or the trace of sinusoidal locomotion tracks (Figure S4). We also examined other behaviors of *sam-4* mutants and detected no defects in mechanosensation, egg-laying, or growth rates. Furthermore, *sam-4* males remain competent to mate. These observations suggest that *sam-4* may encode a specialized neuronal component that promotes efficient SV transport, without being essential for the process.

### SAM-4 encodes a conserved protein expressed broadly in the nervous system

**Figure 2:**
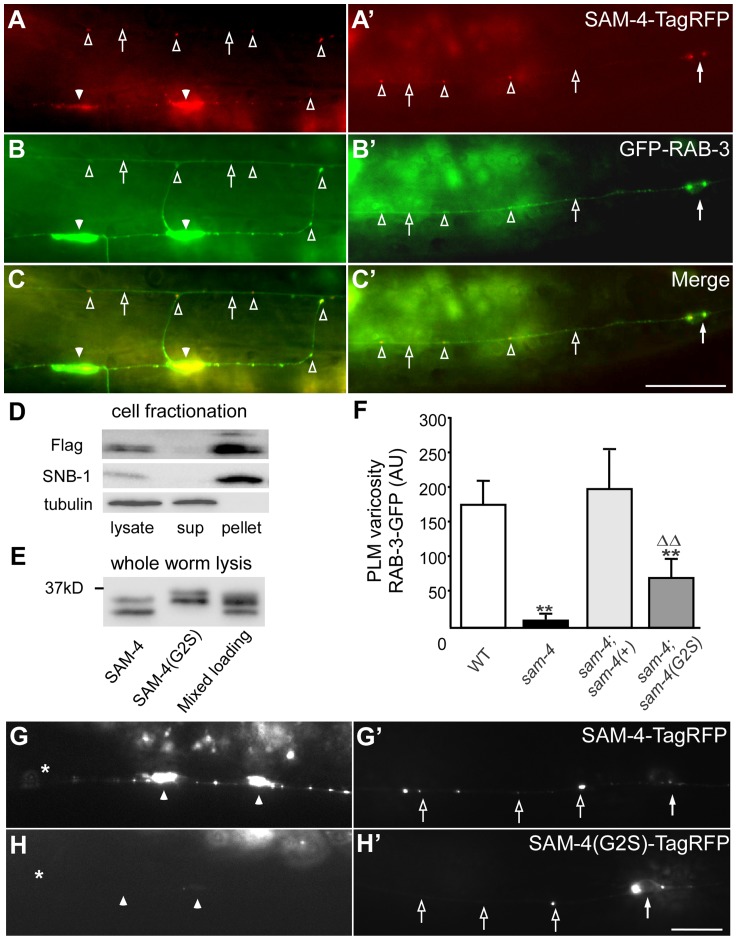
*sam-4* encodes a neuronally expressed protein SV associated protein. (A–C′) Distribution of SAM-4-TagRFP expressed in mechanosensory neurons in the synaptic varicosities (A) and the PLM soma and proximal section of the PLM neurite (C). GFP-RAB-3 localization is also shown in (B and B′) along with the merge (C and C′). (D–E) Western blots of fractionated whole worm extracts (D) and non-fractionated lysis (E) derived from animals expressing 3XFlag tagged SAM-4(+) and SAM-4(G2S) myristoylation mutant proteins probed for SAM-4, the synaptic vesicle protein SNB-1, and the cytosolic protein β-Tubulin. Note the shift in migration of the SAM-4(G2S) mutant. (F) Quantification of GFP-RAB-3 levels in PLM synaptic varicosities. **, P<0.001, relative to wild type; ΔΔ, P<0.001, relative to *sam-4*. Transgenes tested: *jsIs1188* for *sam-4(+)*, *jsIs1265* for *sam-4(G2S)*. Allele tested: *sam-4(js415)*. (G–H′) SAM-4(G2S)-TagRFP (*jsEx1256*) levels are reduced in PLM synaptic varicosities (G and H) but increased in PLM soma (G′ and H′), relative to SAM-4-TagRFP (*jsIs1156*). Solid arrow: PLM soma; open arrow: PLM neurite; solid arrowhead: PLM synaptic varicosity; open arrowhead: florescence marker puncta in PLM neurite; asterisk: vulva. Scale bars: 10 µm.

Positional cloning and transgenic rescue identified *sam-4* as the *C. elegans* gene *F59E12.11* (Figure S5 and Materials and Methods for details), which encodes an evolutionarily conserved 240 amino acid protein (Figure S5) with no identifiable domain structure. An additional open reading frame (ORF) was identified in the 5′ UTR of the *sam-4* mRNA, but these sequences are not required for functions we describe for *sam-4* herein (see discussion for details). SAM-4 is the *C. elegans* ortholog of human LOH12CR1 that was identified as a candidate tumor suppressor based on frequent deletion of this region of human chromosome 12 in acute lymphoblastic leukemia [35]. To confirm the *sam-4* gene identification, we expressed a 3X-FLAG-tagged derivative of the SAM-4 protein (Figure S5C) under its native promoter using a MosSCI strategy [45], [46]. The single copy *sam-4-3XFlag* transgene completely rescued the Sam phenotypes of *sam-4* mutants (Figure 2E). Immunohistochemical analysis of the transgene revealed that SAM-4 is localized primarily to the nerve ring region of the head (Figure S6A), indicating that *sam-4* is broadly expressed in the nervous system.

The *sam-4* mutations we characterized are recessive and likely represent null alleles of *sam-4*. The *js415* allele isolated in our screen introduces a CAA>TAA nonsense lesion at Gln104 (Figure S5A and S5B). *tm3828*, another *sam-4* allele isolated by the Japanese National Bioresource Project, deletes 149 bp of *sam-4*. This deletion removes exon sequences coding for amino acids from Leu66 to Ala100 and results in a frame-shift (Figure S5A and S5B). *tm3828* and *js415* exhibit indistinguishable GFP-RAB-3 mis-accumulation phenotypes (Figure S2). Since both mutations result in severe disruption of coding potential of *sam-4* and have similar phenotypes, we conclude that both alleles represent null mutations.

### SAM-4 is an SV associated protein

To characterize the role of SAM-4 protein in regulating SV transport, we assayed its function when expressed in different cell types (Figure S5C). We found that *sam-4* expression in PLM neurons driven by the *mec-7* promoter rescued the SV accumulation defects in PLMs (Figure S5D) while its expression in PLM postsynaptic partners driven by the *glr-1* promoter did not. These data suggest that SAM-4 functions cell-autonomously to regulate SV transport.

We next used a functional *sam-4-TagRFP* transgene expressed in PLMs to further examine the sub-cellular localization of SAM-4. We observed that SAM-4 preferentially accumulates in the synaptic varicosities of PLMs and small quantities of SAM-4 accumulate as puncta in the neurites (Figure 2A and 2A′), a pattern similar to the GFP-RAB-3 marker (Figure 2B and 2B′). Further examination demonstrated that these SAM-4 particles co-localize well with the RAB-3 labeled SV particles (Figure 2C and 2C′) and furthermore the RAB-3 and SAM-4 particles move together (Figure S6B, Movie 1–3). In addition, SAM-4-TagRFP is retained in the cell body in *unc-104* mutants as previously demonstrated for many other SV proteins including RAB-3 [39], [47]. These observations suggest that SAM-4 may function as a component of the SV trafficking machinery.

To determine if SAM-4 is a SV component, we examined SAM-4 subcellular localization using cell fractionation analysis. We lysed *sam-4-3XFlag* transgenic animals under detergent free conditions, cleared the lysate of large membrane organelles, cytoskeleton, and cell debris using a 15K g spin, then fractionated the extract into a membrane containing 150K g pellet and a cytosolic fraction. We observed that, like the SV protein synaptobrevin SNB-1, SAM-4 was present in the SV membrane-containing pellet but was absent from the cytosolic fraction (Figure 2D), indicating that SAM-4 is likely associated with SVs. Bioinformatic analysis predicts that SAM-4 contains a conserved myristoylation site at its amino terminus (Figure S5B). We then tested if SAM-4 localizes to membranes through the myristoylation signal. Myristoylated proteins typically migrate faster than their non-myristoylated counterparts [48]. We observed a decrease in mobility of SAM-4(G2S)-3XFLAG tagged protein compared to the SAM-4-3XFLAG control expressed at endogenous levels consistent with the hypothesis that this mutation disrupts SAM-4 myristoylation *in vivo* (Figure 2E). However, fractionation of SAM-4 to the membrane compartment was not altered by the SAM-4 (G2S) lesion suggesting that SAM-4 associates with membranes independently of myristoylation (Figure S6C). In fractionation experiments when EDTA and EGTA were omitted from the buffer, we also observed FLAG immunoreactive band 4 kD smaller than the full length SAM-4 which fractionated partially into the cytosol (Figure S6D) suggesting the SAM-4 N-terminus contains a site mediating interactions with an unidentified SV membrane component.

We further examined functional activity of the *sam-4(G2S)* myristoylation mutant and found that the endogenous expression level of *sam-4(G2S)* (*jsIs1265*) only partially rescue *sam-4(null)* mutants (Figure 2F). In addition, we observed that the SAM-4 (G2S) protein is not efficiently delivered to synapses and is largely retained in the soma (Figure 2G–H′). Taken together, these results argue that SAM-4 functions as a SV component to regulate SV trafficking.

### SAM-4 functions as a component of the UNC-104 mediated SV transport machinery

**Figure 3:**
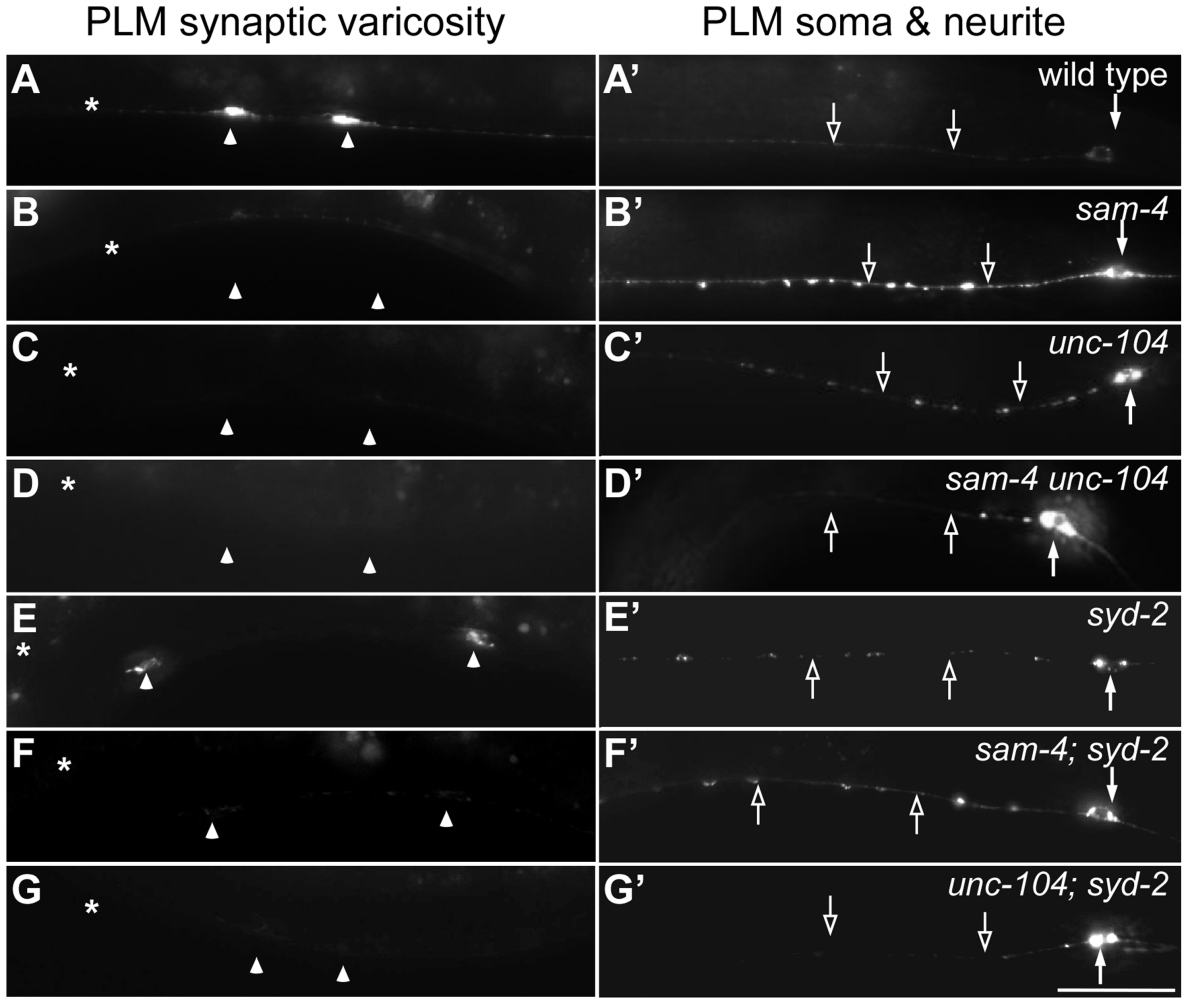
*sam-4* and *syd-2* interact with *unc-104* in transporting SVs. GFP-RAB-3 (*jsIs821*) distribution in PLM neurons of wild type and mutant animals as indicated. A–G panels are focused on the PLM synaptic varicosities and A′–G′ panels are focused on the PLM soma and the proximal portion of the neurite. Alleles tested: *sam-4(js415)*, *unc-104(js901)* and *syd-2(ok217)*. Arrowheads: synaptic varicosities; solid arrows: PLM soma; open arrows: PLM neurites; asterisk: vulva. Scale bar: 20 µm.

**Figure 4:**
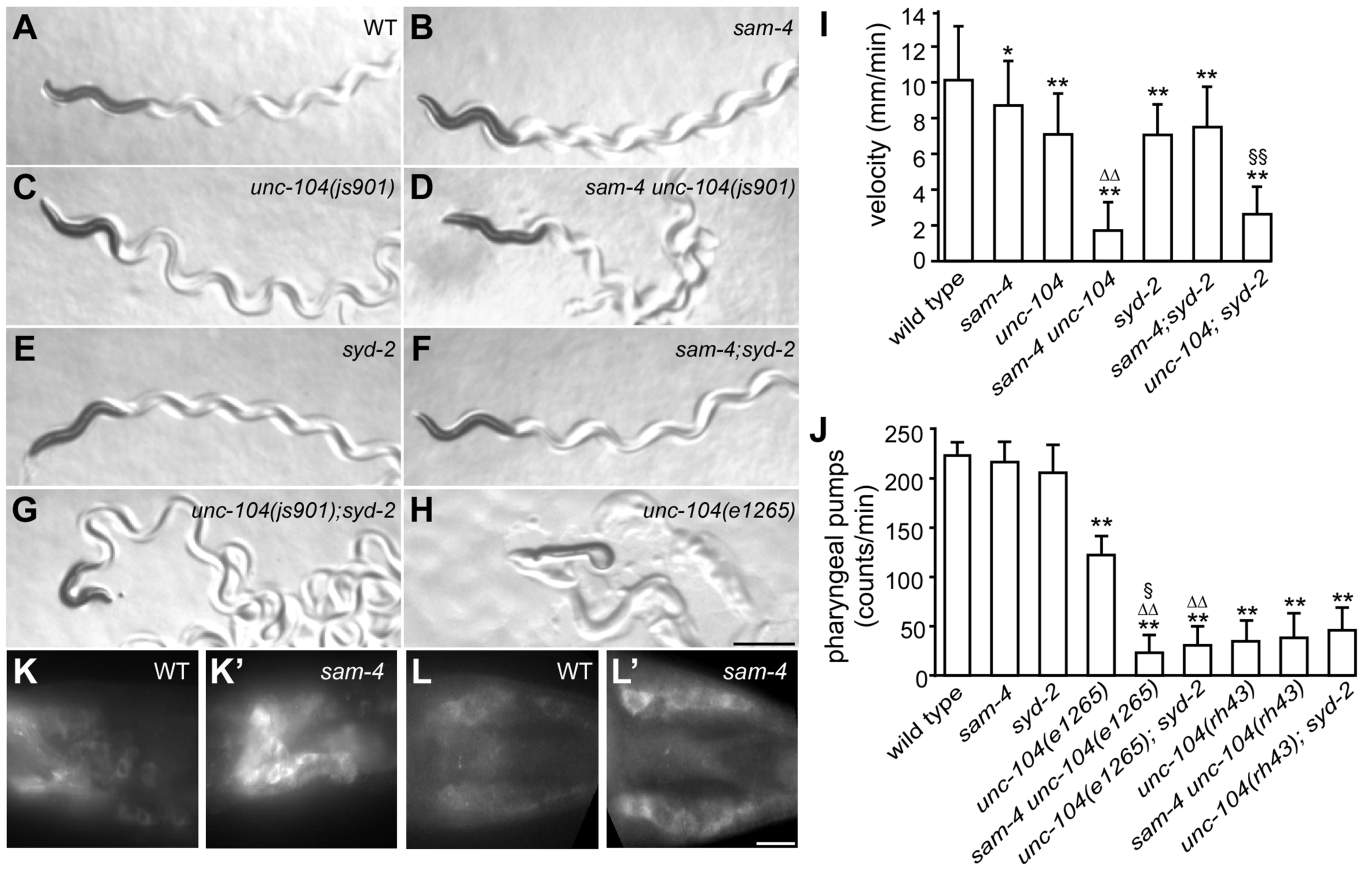
*sam-4* and *syd-2* interact with *unc-104* to regulate behavior. (A–H) Posture and sinusoidal tracts of free moving animals on an *E. coli* lawn. Scale bar 1 mm. (I) Locomotion velocity measurements of wild type and mutant animals. *P<0.05, relative to wild type; **, P<0.001, relative to wild type; §§, P<0.001 relative to *syd-2* and *unc-104*; P<0.001, ΔΔ relative to *sam-4* and relative to *unc-104*. n>20 per group. (J) Pharyngeal pumping assays of wild type and various mutant and double mutant combinations. **, P<0.001, relative to wild type; ΔΔ, P<0.001, relative to *unc-104(e1265)*; §, P<0.05 relative to *sam-4 unc-104(rh43)*; n>25 per group. (K–L′) anti-UNC-104 whole mount immunohistochemistry staining of the head (K and K′) and the tail (L and L′). Scale bar 5 µm. Alleles tested: *sam-4(js415)*, *unc-104(js901)* and *syd-2(ok217)* unless otherwise indicated.

Axonal transport of SVs in synapses is mediated by anterograde transport (largely the KIF1A motor system) and retrograde transport (the dynein motor system). To understand mechanisms by which SAM-4 regulates SV trafficking, we examined genetic interactions of *sam-4* with mutations in both *unc-104* and the dynein heavy chain gene *dhc-1*. Hypomorphic mutations were used because null mutations in both genes are lethal and because point mutations in different domains of UNC-104 are available for analysis. We first tested if SAM-4 is involved in regulating the UNC-104 transport machinery by examining *sam-4 unc-104* interactions. We previously isolated a hypomorphic *unc-104* loss-of-function (lf) mutant, *js901*, with a G1466V lesion in the cargo binding PH domain of UNC-104 that displays very similar phenotypes to *sam-4* (materials and methods for details). These mutants show decreased GFP-RAB-3 levels in PLM synaptic varicosities and increased accumulations in the proximal portion of PLM neurites (Figure 3A–3C′). Furthermore, they displayed mild locomotion defects (Figure 4A, 4C and 4I) while remaining grossly normal in PLM neurite morphology, growth rate and egg-laying behavior. *js901* males remained competent to mate. Overall, the phenotypic defects of *unc-104(js901)* are mild compared to other *unc-104* alleles such as the *e1265* PH domain and the *rh43* motor domain lesions which have severe locomotory defects and slow growth rates. If SAM-4 interacts with UNC-104 to regulate SV transport, we reasoned that *sam-4* mutations would exaggerate the mild *js901* defects. Indeed, we observed that SV soma accumulations are further increased in the *sam-4 unc-104(js901)* double mutant relative to either single mutant (Figure 3A–3D′). Additionally, we found that *sam-4 unc-104(js901)* double mutants exhibit very severe locomotion defects relative to either single mutant, exhibiting defects comparable to severe *unc-104* mutants (Figure 4A–4D, 4I). These results suggest that SAM-4 functions in concert with the UNC-104 protein to regulate the SV trafficking.

It has been previously demonstrated that the UNC-104 PH domain functions independently from the motor domain [49], [50]. The motor domain can walk on microtubules independently of the PH domain, and the PH domain can interact with vesicles independently of the motor domain. To assess the mechanistic implications of the genetic interactions between SAM-4 and UNC-104, we examined the allele specificity of these interactions. Specifically, we first examined *sam-4* interactions with a SV binding defective *unc-104* allele, *e1265*, which introduces a missense mutation (D1498N) in the PH domain and causes severe defects in SV binding [51]. Since *e1265* mutants show virtually no detectable GFP-RAB-3 signal in neurites and severe locomotion defects with essentially no sinusoidal movements within the time-frame of our measurements (Figure 4H), we analyzed the *sam-4* and *unc-104(e1265)* interactions by scoring animals for pharyngeal pumping, a behavior which is also controlled by neuronal activity [52]. We found that pharyngeal pumping rates of the double mutants were significantly lower than those of *e1265* animals (Figure 4I). *sam-4 unc-104(e1265)* double mutants also had lower brood sizes and slow growth relative to either single mutants. These results suggest that SAM-4 acts synergistically with the PH domain to regulate SV trafficking.

We then tested how *sam-4* mutations interact with *unc-104(lf)* mutations in the motor domain. *unc-104(rh43)* introduces two missense mutations in the motor domain and results in its motility defect [51]. These mutants exhibit severe locomotion defects again limiting our assay of animal movements. We applied pharyngeal pumping tests to evaluate their interaction. Surprisingly, we found that pharyngeal pumping defects introduced by the *rh43* mutations are not exacerbated by the *sam-4* mutation (Figure 4J). Furthermore, we noticed that while the pumping defects of *e1265* mutants are less severe than those of *rh43* mutants, these defects of *sam-4 unc-104 (e1265)* are more severe than those of *sam-4 unc-104 (rh43)* (Figure 4J). Thus, s*am-4* exhibits allele specific synthetic interactions with PH domain lesions, but not motor domain lesions of *unc-104*. Taken together, these results suggest that SAM-4 functions by improving the UNC-104 motility, and acts in parallel to the UNC-104 PH domain to regulate SV trafficking.

To further explore the notion that SAM-4 enhances UNC-104 movement, we examined UNC-104 motor activity indirectly in *sam-4* mutants by determining the localization of native protein. While UNC-104 protein is barely detectable in soma of wild type animals, we observed a dramatic increase of somatic UNC-104 accumulation in *sam-4* mutants (Figure 4K–4L′). Since UNC-104 expression level is not affected by the *sam-4* mutations (Figure S9B), these data indicate that UNC-104 motility is disrupted.

By contrast with *unc-104*, dynein *dhc-1* mutants exhibit accumulations of GFP-RAB-3 in the distal portion of the anterior PLM neurite presumably due to disruption of retrograde transport, but have largely wild type levels of GFP-RAB-3 in both the PLM soma and synaptic varicosities. We found that *dhc-1(js319); sam-4* double mutants show vestiges of both mutant phenotypes: while GFP-RAB-3 levels are modestly increased in the distal portion of PLM neurites resembling *dhc-1* phenotypes, GFP-RAB-3 levels are greatly reduced in the PLM synaptic varicosities and increased in the proximal portion and soma resembling *sam-4* phenotypes (Figure S7). This combination of phenotypes is similar to that of *dhc-1(js319); unc-104(js901)* animals (Figure S7). We interpret these phenotypes of *dhc-1; sam-4* double mutants as a combination of the *sam-4* and *dhc-1* induced defects in SV transport. Therefore, *sam-4* shows no obvious genetic interactions with *dhc-1*. Taken together, these genetic interactions support the model that SAM-4 regulates SV anterograde transport through UNC-104 motor domain.

### SAM-4 regulates SV transport by improving processivity

**Figure 5:**
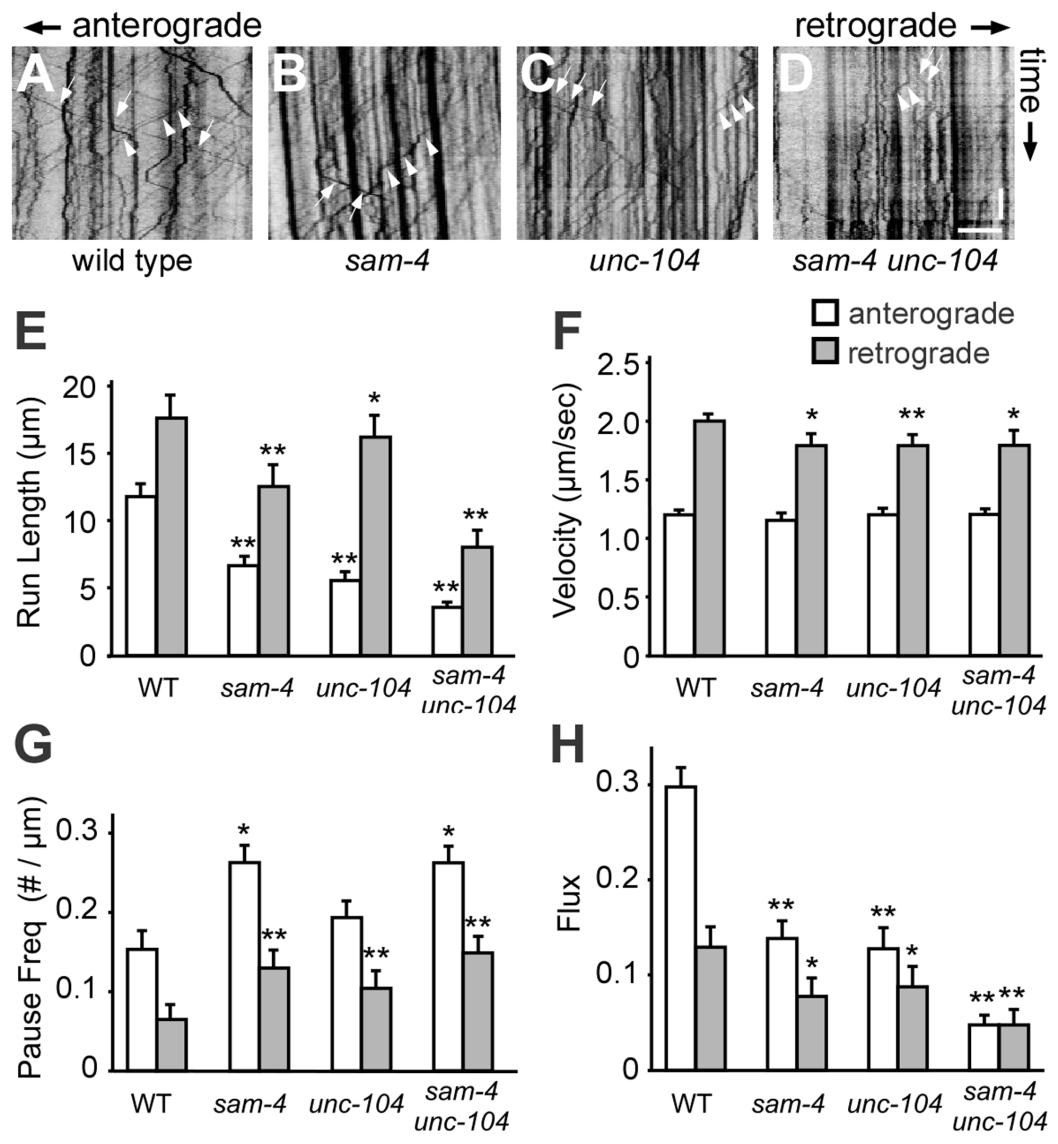
Live imaging of GFP-RAB-3 trafficking in *sam-4* mutants. (A–D) Representative GFP-RAB-3 trafficking kymographs in different genetic backgrounds. Arrowheads: anterograde movements; arrows: retrograde movements. Horizontal scale bar 5 µm; vertical scale bar 5 sec. (E–H) Quantification of anterograde and retrograde SV trafficking in L4 animals. (E) Average mean run length, (F) Average velocity, (G) Average pause frequency, and (H) Average flux are shown. Flux is defined as the number of moving particles per total time. Alleles tested: *sam-4(js415) and unc-104(js901)*. ** P<0.01, ** P<0.001 relative to wild type.

To directly address how SAM-4 regulates SV transport, we examined GFP-RAB-3 puncta dynamics in PLM neurites of *sam-4* mutants using time-lapse imaging (Figure 5). We found that SV anterograde transport is significantly reduced in *sam-4* mutants as revealed by a reduced number of moving particles, reduced run-length of particles and increased frequency of pauses (Figure 5E–5H). However, the velocity of the GFP-RAB-3 transport was similar to wild type (Figure 5F). Retrograde trafficking is similarly affected by *sam-4* mutations (Figure 5), in agreement with previous observations that retrograde trafficking is linked to anterograde trafficking of SVs [51], [53]. *sam-4* defects were similar in severity to those of *unc-104(js901)* mutants. However, *sam-4 unc-104* double mutants show more severe defects in GFP-RAB-3 trafficking (Figure 5), consistent with our behavioral and cell biological observations. These findings argue that SAM-4 regulates the anterograde trafficking of SVs by modulating the processivity of SV transport.

### SYD-2 and SAM-4 regulate SV transport similarly

In *C. elegans*, SYD-2 liprin-α has been shown to regulate SV transport by binding the FHA domain and stalk domain of UNC-104 [34]. With our observations on *sam-4 unc-104* interactions in regulating SV transport, we next examined the relationship between *syd-2* and *sam-4*. We first confirmed that *syd-2(ok217)* null mutants show increased GFP-RAB-3 levels in the PLM soma and decreased levels in PLM synaptic varicosities (Figure 3), suggesting that anterograde SV trafficking is reduced. The GFP-RAB-3 accumulation defects in *syd-2* mutants are less severe than that in *sam-4* mutants (Figure 3). Nevertheless, similar to that observed in *sam-4 unc-104(js901)* mutants, abnormal soma and proximal neurite GFP-RAB-3 accumulations become much severe in *unc-104(js901)*; *syd-2* mutants relative to either single mutant (Figure 3). Furthermore, *unc-104(js901)*; *syd-2* mutants show more severe defects in locomotion than either single mutant (Figure 4I). Similar genetic interactions to those observed in *sam-4 unc-104(e1265)* and *sam-4 unc-104(rh43)* animals were observed in *unc-104(e1265); syd-2* and *unc-104(rh43); syd-2* (Figure 4J). Taken together, these data suggest that SYD-2 acts synergistically with UNC-104 PH domains to regulate SV trafficking in a similar manner as SAM-4.

We next examined *sam-4 syd-2* interactions and observed no detectable genetic interactions between the two mutants. Double mutants display *sam-4*-like GFP-RAB-3 accumulation defects (Figure 3), and similar stimulated locomotion behaviors as either single mutants (Figure 4I). Over-expression of *sam-4* does not suppress *syd-2(ok217)* mutants, and *syd-2(ju487)*, a gain-of-function allele, has no effects on *sam-4* defects (Figure S8). These results are consistent with the hypothesis that SYD-2 and SAM-4 function in the same pathway to regulate SV trafficking.

### *unc-104* gain-of-function mutations suppress *sam-4* and *syd-2* defects

**Figure 6:**
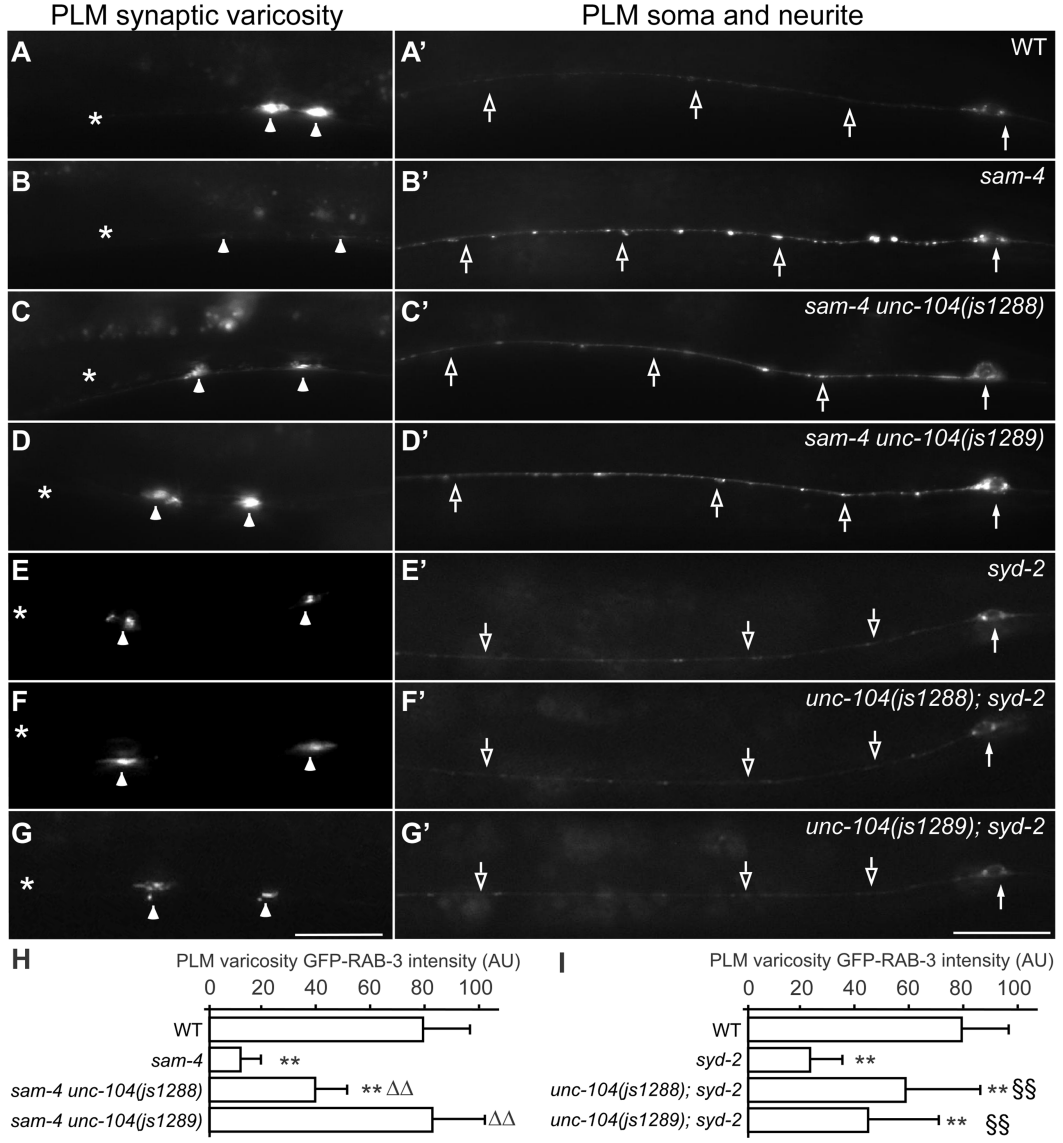
Gain-of-function *unc-104* motor mutations suppress *sam-4* and *syd-2* SV trafficking defects. (A–G′) shown is the distribution of GFP-RAB-3 accumulations in PLM synaptic varicosities (left) and the PLM soma and proximal neurite (right). Arrowheads: PLM synaptic varicosities; solid arrows: PLM soma; open arrows: PLM neurites. Scale bar: 20 µm. (H–I) Quantification of GFP-RAB-3 fluorescence intensity in PLM synaptic varicosities. **, P<0.001 relative to wild type; ΔΔ, P<0.001 relative to *sam-4(js415)*; §§, P<0.001 relative to *syd-2(ok217)*.

**Figure 7:**
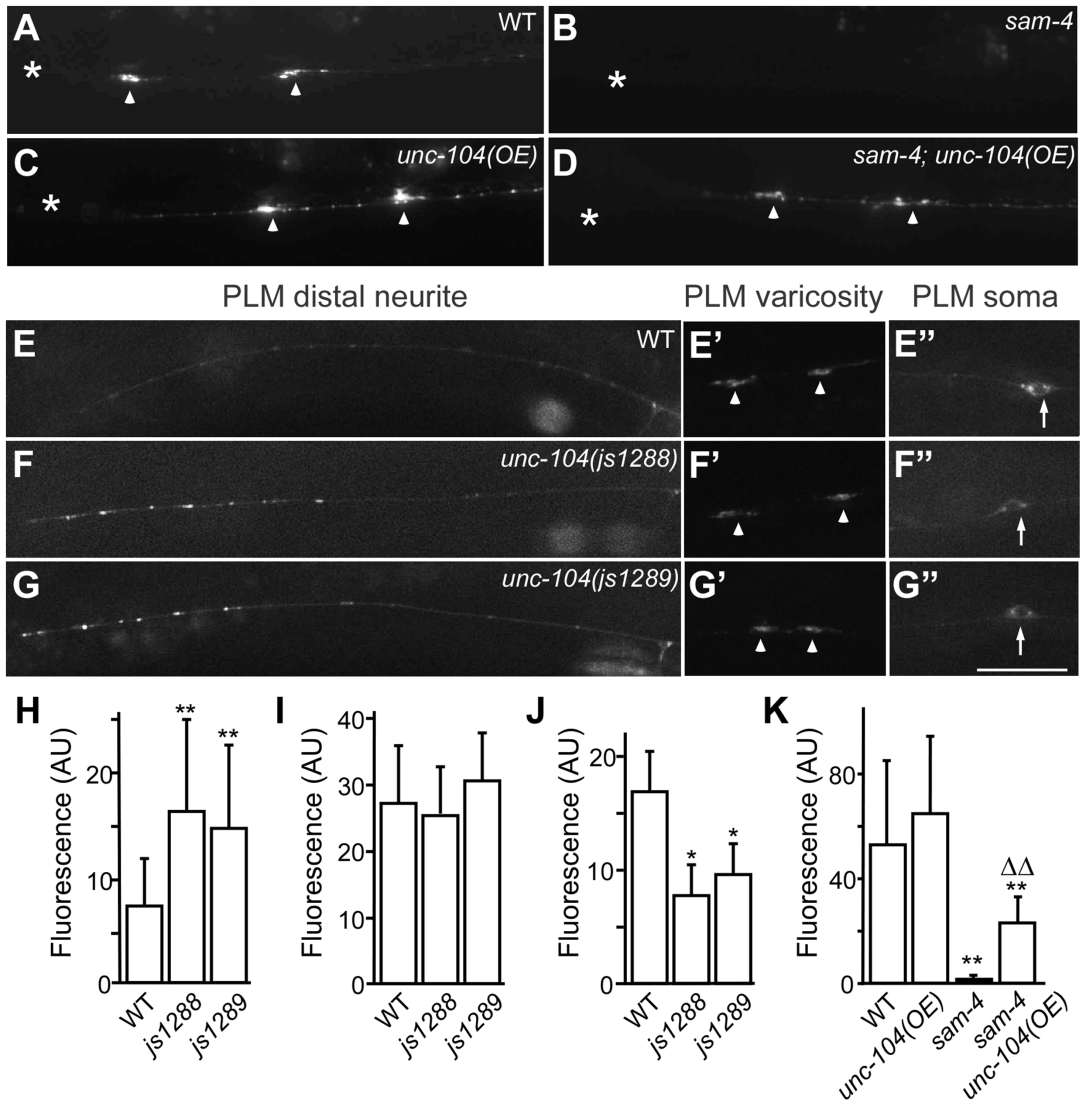
*js1288* and *js1289* are gain-of-function mutations in the motor domain of the *unc-104* gene. (A–D) Effects of *unc-104* overexpression (*OE, jsIs1111*) on TagRFP-RAB-3 (*jsIs1263*) accumulation in PLM synaptic varicosities of wild type (A and C) and *sam-4 (js415)* (B and D). (E–G″) Distribution of the SV marker GFP-RAB-3 in PLM neurons. Shown are representative images of the GFP-RAB-3 (*jsIs821*) signal observed in the distal end of PLM neurites (E–G), the PLM synaptic varicosities (E′–G′), and the PLM soma (E″–G″). Arrow: PLM soma; arrowhead: PLM synaptic varicosity; asterisk: vulva. Scale bar: 20 µm. (H–J) Quantification of GFP- RAB-3 fluorescence intensities in the distal end of the neurite (30 µm) (H), the synaptic varicosities (I), and the soma (J). Note that, due to differences between imaging conditions for individual PLM anatomic regions, arbitrary units and fluorescence intensity between regions are not comparable. (K) Quantification of TagRFP-RAB-3 fluorescence intensity in PLM synaptic varicosities in different genetic backgrounds. *, P<0.01 relative to wild type; **, P<0.001 relative to wild type; ΔΔ, P<0.001 relative to *sam-4(js415)*.

To further understand how SAM-4 activity regulates SV trafficking, we conducted a genetic screen for *sam-4* suppressors. Using ENU induced mutagenesis, we screened mutated progeny of *sam-4(js415); jsIs821* for animals with increased GFP-RAB-3 signal in PLM synaptic varicosities (Figure 6A–6D) and isolated two suppressors from roughly 100,000 genomes screened. Combining traditional genetic mapping and whole genome sequencing strategies, we identified both mutations as novel *unc-104* alleles (see Materials and Methods for details). Interestingly, we found that the alleles introduce missense mutations in the UNC-104/KIF1A motor domain: S211A (*js1288*) and D177A (*js1289*), both of which are conserved in mammalian molecular motor proteins (Figure S9). Further genetic tests showed that both alleles are semi-dominant in suppressing *sam-4* defects. Additionally, we found that over-expression of wild type *unc-104 (unc-104(+))* in PLM neurons suppresses *sam-4* defects (Figure 7A–7D and 7K), but over-expression of *sam-4* does not suppress *unc-104* defects (Figure S7). Similar suppression analysis using *syd-2* mutants by these *unc-104(gf)* mutations also revealed suppression by *unc-104(gf)* alleles (Figure 6). Taken together, these data argue that both *js1288* and *js1289* are gain-of-function alleles of *unc-104*, and *unc-104* is epistatic to *sam-4* and *syd-2*.

**Figure 8:**
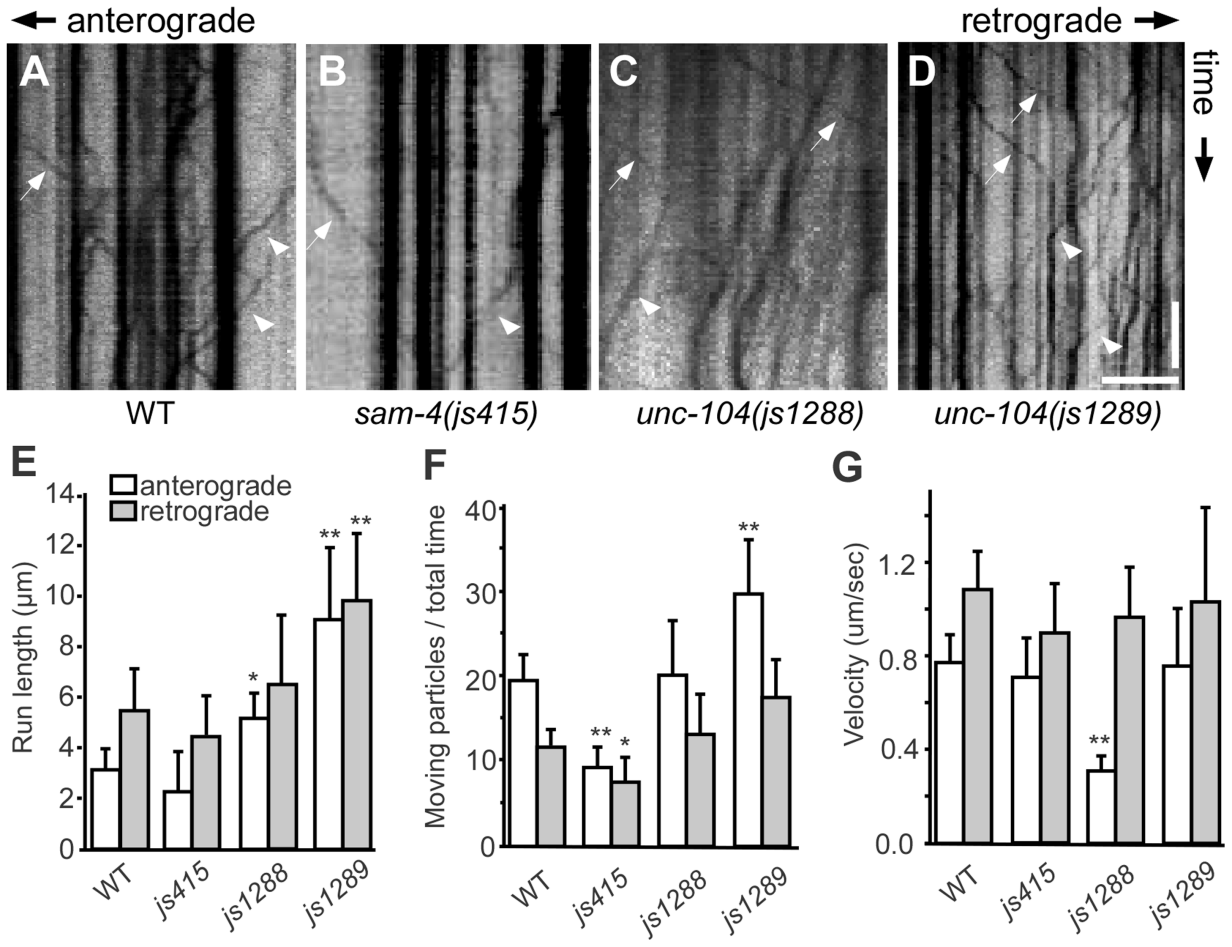
Live imaging of GFP-RAB-3 trafficking in *unc-104* mutants. (A–D) Representative SV trafficking kymographs in different genetic backgrounds. Arrowheads: anterograde movements; arrows: retrograde movements. Horizontal scale bar 5 µm; vertical scale bar 5 sec. (E–G) Quantification of anterograde and retrograde GFP-RAB-3 trafficking in mid-L1 stage animals. (E) Average of run length of GFP-RAB-3 particles, (F) Moving particles observed in 40 sec, (G) Average velocity of moving particles. *, P<0.01 relative to wild type; **, P<0.001 relative to wild type, n = 15.

To address how the *unc-104(gf)* suppresses the SV trafficking defects of *sam-4*, we characterized the two *unc-104* alleles in the absence of *sam-4*. In isolation, *js1288* and *js1289* show grossly normal mechanosensory neuron anatomy (Figure 7F, 7G). We analyzed their effects on transport by examining GFP-RAB-3 distribution *in vivo*. We found that GFP-RAB-3 accumulations are significantly increased in the distal part of PLM neurites (Figure 7E–7H) in each of these *unc-104* mutants but decreased in the soma (Figure 7E″–7G″, 7J), indicating that SV transport is enhanced by these two mutations. However, we did not observe GFP-RAB-3 increase in PLM synaptic varicosities (Figure 7E′–7G′, 7I). This is probably because either SV levels in PLM varicosities are already saturated in the wild type background or other mechanisms exist at pre-synapses to maintain SV homeostasis. To further understand how these mutations affect SV dynamics, we examined GFP-RAB-3 trafficking using live imaging (Figure 8). We found that both mutations result in increased run length of GFP-RAB-3 transport (Figure 8E). We also noticed that *js1289* results in greater flux of GFP-RAB-3 (Figure 8F), while *jsIs1288* reduces SV transport velocity (Figure 8G). Thus, processivity of vesicle transport is increased in both gain-of-function mutants, though perhaps by distinct mechanisms. Western blot analysis of protein levels showed that neither of these two lesions alter UNC-104 protein levels *in vivo* (Figure S9B). Hence, increasing processivity of the SV transport through the UNC-104 motor domain can partially bypass the need for SAM-4. This is consistent with our hypothesis that SAM-4 functions through the UNC-104 motor domain to regulate SV transport.

## Discussion

In this study we have identified the conserved protein SAM-4 as a novel vesicular component regulating SV transport in *C. elegans*. SAM-4 behaves as a SV associated protein and modulates transport probably by regulating the motor domain activity of UNC-104. This possibility is supported by our identification of two *unc-104(gf)* motor domain mutations, which suppress *sam-4* SV transport defects. Although our genetic evidence is consistent with a SAM-4 UNC-104 interaction, we have been unable to detect any evidence for physical interactions between SAM-4 and UNC-104 either *in vitro* by yeast two-hybrid analysis or *in vivo* by co-immunoprecipitation. Therefore, SAM-4 UNC-104 interactions may be mediated by other components. Our genetic data also indicate that SAM-4 acts in the same pathway as SYD-2 in regulating SV transport. We propose a model in which SV-bound SAM-4 regulates SV transport together with SYD-2 through UNC-104, likely via its motor domain (Figure S10).

It is known that SV transport is regulated, but little is known of the molecular mechanisms involved. The identification of SAM-4 as a SV-bound regulator of KIF1A/UNC-104-mediated transport defines a new pathway for modulation of axonal transport. Although SAM-4 is conserved, analysis of the protein sequence revealed only a N-terminal myristoylation motif, which appears to contribute to SAM-4 activity. The lack of identifiable protein domains in the protein make it difficult to speculate on a specific mechanism of action. We have proposed that SAM-4 modulates SV transport processivity by modulating UNC-104 motor activity because we observed strong genetic interaction between *sam-4* and *unc-104* cargo binding mutants but not with motor domain mutants. Furthermore, motor domain *gf* mutations suppress *sam-4* defects arguing that increase of motor processivity can partially bypass SAM-4 activity.

In addition, the genetic interactions between *syd-2* and *sam-4* support a processivity based mechanism of action for SAM-4. Both worm and mammalian Liprin-α/SYD-2 interact with KIF1A/UNC-104 [32], [34] and Liprin-α/SYD-2 is required for efficient SV trafficking in both *C. elegans* and *Drosophila* [33]. Our data argue *syd-2* functions in the same pathway as *sam-4* in regulating SV transport since each null mutant shows very similar interactions with both *unc-104(lf)* and *unc-104(gf)* lesions, but do not display obvious interactions with each other. However, SAM-4 may play a more central role in this process since the SV trafficking phenotypes in *syd-2(null)* are less severe than those of *sam-4(null)*.

In this study, we recovered two gain-of-function mutations in the motor domain of *unc-104* that increase the processivity of the motor in cargo movement assays. Kinesin mediated SV transport is an ATP driven process, which depends on motor-microtubule binding. The ATP hydrolysis catalytic core lays in the switch I region of the KIF1A/UNC-104 motor domain (Figure S9). Lesions (for example H215Y in *unc-104(y211)*, see Figure S1) in this domain cause severe SV trafficking defects. The *js1288* mutation occurs in S211A adjacent to S212 (S215 in mammalian KIF1A) which coordinates the gamma-phosphate of ATP in the ATP-bound crystal structure [54]. Consistent with the hypothesis that this lesion alters rates of ATP hydrolysis, we observed a lowered velocity of transport in *js1288* mutants. However, the biochemical mechanism underlying the increase in processivity is unclear. The other mutation, *js1289*, is a D177A substitution (mammalian KIF1A D180) in loop 8 of the motor domain. Previous studies [54] showed that loop 8 is one of three microtubule binding regions in the motor domain and thus processivity in this mutant could be increased due to changes in the affinity for microtubules. In addition to suppressing SV trafficking defects in *sam-4(null)*, both of these lesions result in increased accumulations of SVs in the distal portion of PLM neurites where no synapses have been seen at the ultrastructural level [40]. Therefore, these *unc-104 (gf)* mutations disturb the normal homeostasis of SV trafficking and thus may not necessarily represent beneficial biochemical modifications. However, the lesions argue strongly that processivity is not optimized in KIF1A and suggest the possibility that KIF1A activity could be modified, for example by pharmacological compounds, in diseases where axonal transport is compromised.

It is worth noting that several lines of evidence imply *sam-4* also regulates other processes in non neuronal cells. First, *sam-4* neuronal phenotypes are partially maternally rescued. Some *sam-4* animals segregating from the *sam-4/+* mother even display wild type levels of GFP-RAB-3 at PLM synapses. Second, *sam-4* is likely post-transcriptionally regulated. The *sam-4* locus is highly unusual (for nematodes) in that it has two 5′ “non-coding” exons (Figure S5). These exons contain a small 79 amino acid ORF. A similar ORF is found in the 5′-end of *sam-4* in highly divergent nematodes and the synonymous codon usage in these nematodes indicates it is being selected as coding sequence (Figure S11). The ORF is homologous to the APC13, a small subunit of the Anaphase Promoting Complex (APC) which was previously described for plant parasitic nematodes [55], but recognized in model system databases. We, and other investigators, observed lethality when performing RNAi against *sam-4* even though both nonsense and deletion alleles of *sam-4* are fully viable. These *sam-4* RNAi lethal phenotypes are similar to those of other APC complex component genes. These include defects in meiosis in the early embryo [56], oocyte deformation and sterility [57] and failure to segregate germline P-granules [58]. We posit the lethality phenotype associated with *sam-4* RNAi is likely due to reduced expression of this upstream ORF encoding an APC13 homolog.

The APC complex plays critical roles in regulating progression through the cell cycle. However, recent work has also highlighted several critical roles for APC complexes in neuronal development [59]. In particular, disruption of the APC complex alters axon growth, post-synaptic glutamate receptor levels [60] as well as the size and number of presynaptic boutons [61]. Interestingly, in regulating bouton number in *Drosophila*, the APC complex works in conjunction with liprin-α. Thus, the APC complex, SAM-4 and liprin-α appear linked at multiple different regulatory levels. Further investigations of the non-neuronal roles of SAM-4, the role of the APC13 encoding upstream ORF in regulating SAM-4 expression, and the potential role of the APC complex in regulating axonal transport are clearly warranted.

Although SAM-4 is evolutionarily conserved, no human disease conditions have been specifically associated with lesions in human *sam4* (LOH12CR1), a gene within a region often deleted in acute lymphoblastic leukemia. Notably, worm *sam-4* mutants display virtually indistinguishable phenotypes from mild *unc-104/KIF1A* mutants and human diseases are associated with KIF1A. Specifically, motor domain lesions (A255V and R350G) in human KIF1A underlie the molecular basis of the rare recessive late onset spastic paraplegia SPG30 [6] and a frameshift mutation in the PH domain underlies a form of hereditary sensory and autonomic neuropathy [5]. Further genetic and biochemical studies of SAM-4 in both invertebrates and vertebrates will be required to define the underlying biochemical mechanisms as well as physiological inputs that modulate SAM-4 action in regulating axonal transport.

## Materials and Methods

### Strains and genetics

*C. elegans* animals were maintained using standard methods [62]. All strains used except for those used for SNP mapping were derivatives of the Bristol N2 wild type background. Animals were grown at the room temperature (22.5°C), unless specified. Strains used are listed in Table S1. The genotype of strains was confirmed by PCR using oligonucleotides listed in Table S2.

### Transgene integration

*jsIs1238 II*, *jsIs1156 IV*, *jsIs1263 IV*, *jsIs1188 IV* and *jsIs1189 IV* transgenes were integrated using MosSCI with EG4322 for integration on chromosome II and EG5003 for integration on chromosome IV [45], [46] and confirmed to be single copy by long range PCR amplification. *jsIs1073 and jsIs1075* were generated using a bombardment protocol with *Cbunc-119* as the integration marker [63].

### *sam-4* molecular cloning

Genetic three-factor mapping narrowed the *sam-4* mutation to an interval between *dpy-25* and *rol-6* on chromosome II. Single nucleotide polymorphism (SNP) mapping was used to position *sam-4* with CB4856 as a reference strain and narrowed the mutation down to a 163 kb region on Chromosome II between the SNPs CE2-141 and pkP2147. This region is covered by 5 fosmids. Using germline transformation rescue tests, we further mapped *sam-4* down to the fosmids WRM0610dH02 and WRM0632aA08. The *sam-4(js415)* lesion was identified by candidate gene sequencing in this region. A C>T nucleotide change was detected in the second exon of the predicted gene F59E12.11. *sam-4* defects are fully rescued by a transgene expressing the hypothetic F59E12.11 gene, which is predicted to encode a 240 amino acid protein. These data identify F59E12.11 as *sam-4*.

### Isolation of *unc-104* mutants

*unc-104(js901)* was isolated in a non-clonal forward screen for mutations that mislocalized RBF-1-GFP *(jsIs423)*. L4 *jsIs423* animals were mutagenized using 50 mM ethyl methanesulfonate (EMS) for 4 hrs and placed on *E. coli* seeded agar plates. F2 animals derived from these animals were screened for mislocalization of GFP from the nerve ring to cell bodies surrounding the nerve ring. *js901* was mapped to chromosome II by classical genetic mapping strategy, and tested for non-complementation with *unc-104(e1265)*. The entire coding sequence of *unc-104* was sequenced in *js901* revealing a GGA to GTA that changes Gly1465 to Val. This lesion resides within the PH domain of UNC-104.

*unc-104(js1288)* and *unc-104(js1289)* were isolated in a screen for suppressors of the PLM synaptic varicosity phenotype of *sam-4*. N-ethyl-N-nitrosourea (ENU) mutagenesis was performed using standard methodology [64]. Briefly, *sam-4(js415)* animals were treated with 0.6 mM ENU for 4 hours at the room temperature. Treated animals (P0) were transferred to fresh food (10 L4s or young adults per 100 mm plate). P0 animals were removed from the plates 24–48 hours later. F1 animals were counted 2–3 days later to estimate number of mutagenized chromosomes screened. The F2 animals were screened for increased GFP-RAB-3 signal in PLM varicosities using a fluorescent dissecting microscope. The suppressors displayed tight linkage to *sam-4*. Phenotypic analysis of *sam-4(js415) js1288/sam-4(js415)* and *sam-4(js415) js1289/sam-4(js415)* revealed both were semi-dominant suppressors of the Sam phenotype. Sequencing of the *sam-4* coding region revealed no mutation in *sam-4* gene of these isolates. *js1289* was mapped to between *sam-4* and *rol-6* by three factor mapping, crossing *lin-31(n301) sam-4(js415) js1289 rol-6(187)*; *jsIs821* to CB4856 and screening for Lin Sam non-Rol and Lin Sam Sup (suppressor) non-Rol recombinants 18 of 21 recombinants had recombination events between *sam-4* and *js1289* and 3 between *js1289* and *rol-6*. 100 bp paired-end whole genome sequencing of homozygous strains was conducted at Oklahoma Medical Research Foundation. The data were analyzed using Whole Genomes, a web-based alignment and analysis program, and revealed lesions in *unc-104*: An Asp177 to Ala (GAC to GCC) lesion in *js1288* and a Ser211 to Ala (TCA to GCA) lesion in *js1289*.

### Molecular biology

Plasmid DNA clones were constructed using standard molecular biology techniques.

#### NM2132 (*sam-4* genomic clone)

A *sam-4* full-length genomic fragment was PCR amplified from the fosmid clone WRM068cE02 using oligonucleotides 3645 and 3646, digested with KpnI/PstI and ligated into KpnI/PstI digested pBluescript SK(+). The *sam-4* coding region in this plasmid was confirmed by DNA sequencing.

#### NM2348 (*sam-4::3XFlag*)

A 3XFlag tag was introduced at the C-terminus of SAM-4 by PCR amplification using oligonucleotides 3985 and 3986 and recircularized using In-Fusion cloning kit (Clontech Labs Inc.).

#### NM2347 (pCFJ178 sam-4-3XFlag)

NM2348 was digested with PstI and KpnI and a *sam-4*::3XFlag fragment was inserted into PstI KpnI digested pCFJ178 [45].

#### NM2364 (sam-4(G2S) genomic clone)

NM2132 was mutagenized using a DpnI mediated mutagenesis protocol [65] using oligonucleotides 4059 and 4060.

#### NM2662 (pCFJ178 sam-4(G2S)-3XFlag)

A PshAI/KpnI fragment containing the N-terminus of SAM-4 was ligated into PshAI/KpnI digested NM2347.

#### NM2057 (*mec-7p-tagRFP-mito unc-119*)

TagRFP-mito from pTagRFP-mito (Evrogen) was digested with NheI, filled in, digested with EagI and inserted into NM2041 (Kumar et al. 2009) digested with SalI, filled in, and then digested with EagI replacing GFP-ELKS-1 with Tag-RFP-mito.

#### NM2066 (*mec-7p TagRFP-ELKS-1 unc-119*)

was constructed by amplifying TagRFP from pTagFRP-mito (Evrogen) using oligonucleotides 3571 and 3572, digesting the purified PCR product with NcoI and BsiWI and replacing the GFP in NM2041 excised with NcoI and BsrGI.

#### NM2173 (*mec-7p::sam-4-TagRFP*)

*sam-4* genomic sequences were amplified by PCR using oligonucleotide 3701 and 3702, digested with NheI and NcoI, and inserted into NheI-NM2057 replacing the N-terminal mitochondrial tag with *sam-4* sequences.

#### NM2238 (*mec-7p::sam-4-TagRFP*)

The BssHII/SpeI fragment of NM2173 containing *mec-7p::sam-4-TagRFP* was inserted into BssHII/XbaI digested pCFJ178.

#### NM2351 (*pCFJ178 glr-1p::sam-4-TagRFP*)

The *glr-1* promoter was amplified by PCR using oligonucleotides 3981 and 3984, digested with SphI and NheI and ligated into SphI/NheI digested NM2238).

### Light microscopy

Transgenic animals were imaged using epi-fluorescence on an Olympus BX60 equipped with an X-CITE120 mercury lamp (EXFO) using standard GFP and RFP filter sets. Images were taken with a Retiga EXi CCD camera using OpenLab software and processed using Adobe Photoshop.

### Transgene integration

*jsIs1238 II*, *jsIs1156 IV*, *jsIs1263 IV*, *jsIs1188 IV* and *jsIs1189 IV* transgenes were integrated using MosSCI with EG4322 for integration on chromosome II and EG5003 for integration on chromosome IV [45] with modifications. These transgenic lines were confirmed to be single-copy integration events by long range PCR (Details available online: http://thalamus.wustl.edu/nonetlab/ResourcesF/Resources.html). *jsIs1073* and *jsIs1075* lines were generated by integrating NM2057 and NM2066 using a bombardment protocol with *Cbunc-119* as the integration marker [63].

### Locomotion assays

Animals were assayed at the room temperature on NGM agar. L4 animals (or as indicated) were transferred to a bacteria-free plate to allow them clear off bacteria (2–3 min). Subsequently, these animals were transferred to another bacteria-free plate and imaged immediately for 10–20 sec. Animal movements were recorded using LG-3 frame grabber run by ScionImage software at 1 frame/sec for 40 images total. These recordings were then analyzed using wormtracker plus [66]. Only animals in the imaging field>14 consecutive frames recorded were used in the velocity analyses.

### Pharyngeal pumping assays

L4 animals on bacterial lawns of OP50 on NGM agar were scored at the room temperature. Pharyngeal pumping rates was determined by counting contractions of the terminal bulb for 1 minute per animal.

### Immunostaining

Immunohistochemistry and western blots were performed as previously described [67], [68]. For FLAG immunohistochemistry staining, animals were grown at room temperature and fixed in methanol/acetone. For SAM-4-3XFlag fractionation, 0.5 mM EGTA and 0.5 mMEDTA were added in the fractionation buffer as indicated in Figure S6. Mouse anti-Flag (1∶200, Sigma, Cat. A8592) primary antibody incubations were performed overnight at 4°C. Alexa conjugated secondary antibodies (Invitrogen) were incubated for 2 hrs at room temperature at 1∶500. Antibody used for western blots: anti-FLAG (1∶1000); anti-β-tubulin (1∶1000, E7, Developmental Studies Hybridoma Bank, Iowa city), anti-UNC-104 (1∶40) [51].

### Live imaging and analysis of SV transport dynamics

For young adult animals (used in Figure 5), hermaphrodites were immobilized with 3–5 mM levamisole (Sigma-Aldrich) in M9 buffer and mounted on a 2% agarose pad. Time-lapse images of GFP-RAB-3 were acquired and analyzed as described before [51]. The numbers of moving particles in a 15–20 µm region at a distance of 15–25 µm away from the PLM soma were used for flux calculations.

For mid-L1 staged animals (20–24 hrs after hatch, used in Figure 8), we used an anesthetic-free protocol to image GFP-RAB-3 [69]. Specifically, animals were immobilized in 0.5 µl of 0.10 microspheres (Cat# 00876, Polysciences, Inc.) on 10% agarose pads. Time-lapse imaging was acquired using 100×/1.30 oil objective on a Axioskop (Zeiss) equipped with ASI piezo XYZ-motorized stage, Sutter instruments high speed electronic filter wheels and shutters, and a Hamamatsu Orca-R2 cooled CCD camera all controlled by Volocity software (PerkinElmer Inc.). Time lapse images were acquired for 40 seconds at a speed of 5 frames per second with an exposure time of 200 ms. Particle dynamics were analyzed with Volocity software. Total moving particles were counted in the 35 µm region at a distance of 20 µm away from the PLM soma.

To record GFP-RAB-3 co-movements with SAM-4-TagRFP, confocal images were collected with a Hamamatsu Flash 4.0 CMOS camera attached to a Yokogawa Spinning Disc Confocal apparatus on an Olympus IX73 inverted microscope. 0.33 sec Green and Red channels exposure were taken consecutively, and captured at 1 sec intervals, and image series were assembled into movies using Micro-Manager software (available at micro-manager.org).

### Statistics

P values were determined using GraphPad Prism. Multi-group data sets were analyzed by a one-way ANOVA with post-hoc Holm-Sidak's test for multiple comparisons. A t-test was used for paired data sets.

## Supporting Information

Figure S1 SV trafficking in PLM neurons in *unc-104* mutants. (A) Diagram of *C. elegans* mechanosensory system anatomy. (B–D′) Distribution of GFP-RAB-3 (*jsIs821*) accumulations in PLM synaptic varicosities (B, C and D) and PLM soma (B′, C′ and D′) in L4 wild type and *unc-104* mutants. The D1497N PH domain *unc-104(e1265)* mutant is homozygous viable but severely uncoordinated. The H215Y motor domain *unc-104(y211)* mutant is sub-viable. Most animals arrest as L1 larvae, but under optimal growth conditions (moist plates at 15°C), a subset of animals eventually reach adulthood and occasionally produce progeny. However, the strain is difficult to maintain as a homozygous stock. Arrowheads: PLM synaptic varicosities; arrows: PLM soma; carets: PVM soma; scale bar: 20 µm.
(JPG)

Figure S2 Temperature sensitivity of *sam-4(js415)* and *sam-4(tm3828)*. Animals were grown at different temperatures as shown and the GFP-RAB-3 signal (*jsIs821*) in L4 animals was imaged under the same illumination and camera settings. Arrowheads: PLM synaptic varicosities; solid arrows: PLM soma; open arrows: PLM proximal neurites; asterisk: vulva. Scale bar: 20 µm.
(JPG)

Figure S3 SV distribution in SAB motor neurons. (A) Diagram of the anatomy of *C. elegans* SAB neurons. (B and C) SNB-1-GFP distribution in SAB neurons of *jsIs42 (unc-4p::snb-1-GFP)* in wild type (B) and *sam-4(js415)* (C) animals. Bracket: distal region of the SAB neurites; open arrows: proximal region of SAB neurites; solid arrows: SAB soma. (D and E) SNB-1-GFP distribution in ventral nerve cord neurons of *jsIs1 (snb-1p::snb-1-GFP)* in wild type (D) and *sam-4(js415)* (E) animals. (F and G) GFP-RAB-3 distribution in ventral nerve cord neurons of *jsIs682 (rab-3p::GFP-rab-3)* in wild type (F) and *sam-4(js415)* (G) animals. Solid arrowheads: neuron soma; asterisk: vulva. Scale bar: 20 µm. (H) Western blot of fractionated worm lysis for SV protein SNB-1 and cytosolic marker GAPDH in wild type and *sam-4* animals (See Results for cell fractionation details).
(JPG)

Figure S4 Behavioral defects of *sam-4(js415)* mutants. (A) Stimulated velocity of moving animals of different ages grown at 25°C. L4 animals were transferred to fresh food for tests at day 0 (**: P<0.001). (B–E) Posture of wild type and *sam-4* animals 1 day (B and C) and 3 days (D and E) after molting to the adult stage.
(JPG)

Figure S5 Molecular genetic characterization of the *sam-4* gene. (A) Diagram of the genomic organization of the *dpy-25- rol-6* region of chromosome II and the structure of the *sam-4* gene with genetic lesions shown. The *tm3828* junction fragment is caaaatgttcttgtaatcgtgttgΔaaaacggggaatttcggtgaatctt. The *js415* is a C>T transition in the following sequence actagcaacacgatta[C/T]aagaacattttgcc. (B) Alignment of SAM-4 with its orthologs in fly, mouse and human. The conserved N-terminal myristoylation consensus sequence (MGXXX[S/T] [48]) and molecular lesions in *js415* and *tm3828* are indicated. (C) Diagram of *sam-4* transgene constructs used in analyzing SAM-4 activity. *sam-4* exons are in black and untranslated regions of the message in gray. Distinct promoters, fluorescent proteins, molecular tags, and 3′ UTRs are color coded in the diagram. (D) Defects of SV accumulation in PLM synaptic varicosities in *sam-4(js415)* mutants were rescued by expression of *sam-4* transgene cell autonomously. Arrowheads: PLM synaptic varicosities; arrows: PLM soma; asterisk: vulva; scale bar: 20 µm.
(JPG)

Figure S6 Localization of SAM-4. (A) Anti-FLAG immunohistochemistry of a single copy *sam-4-3XFlag* transgene (*jsIs1188*) expressed under the *sam-4* promoter in *sam-4(js415)*. Expression was detected in the nerve ring (arrow). Scale bar: 20 µm. (B) Co-localization of moving GFP-RAB-3 (*jsIs821*) and SAM-4-TagRFP (*jsIs1156*) particles in a PLM neurite. Recorded fragment is 30 µm away from the PLM soma. Anterograde is to the left. Note that the SAM-4-TagRFP particle is just ahead of GFP-RAB-3 particle due to time lapse during acquiring fluorescent signal alternatively: at each time point, the GFP signal was captured first for 0.33 sec followed by RFP for 0.33 sec. Scale bar: 1 µm. (C–D) Western blots of cell fractions of different genotypes as indicated with EGTA EDTA containing (C) and with non-EGTA/EDTA containing (D) buffer.
(JPG)

Figure S7 Genetic interactions of *sam-4* and *unc-104* with *dhc-1*. Distribution of GFP-RAB-3 accumulations in the PLM soma (A–F), the distal (most anterior) portion the PLM neurite (G–L) and the PLM synaptic varicosities (M–R) in different genetic backgrounds as indicated. Alleles tested: *sam-4(js415)*, *dhc-1(js319)* and *unc-104(js901)*. Scale bar: 20 µm.
(JPG)

Figure S8 Gain-of-function genetic tests on *sam-4(js415), syd-2(ok217) and unc-104(js901)* mutations. A–G) Shown is the distribution of GFP-RAB-3 accumulations in PLM synaptic varicosities in different genetic backgrounds. *sam-4* over expression (*sam-4(OE)*) was examined using a *sam-4* transgene (*jsIs1156*) expressed in PLM neurons, while *syd-2(ju487)* was used for *syd-2* gain-of-function tests. Alleles used: sam-4(js415) and *unc-104(js901)*.Arrowheads: PLM synaptic varicosities; asterisk: vulva. Scale bar: 20 µm.
(JPG)

Figure S9 Characterization of UNC-104 motor domain mutants. (A) KIF1A/UNC-104 alignment focusing on the motor region containing the mutated residues in our isolated *unc-104* alleles and the ATP catalytic core as indicated. (B) Western blot for UNC-104 expression levels in different genetic backgrounds (left panel) and its quantification summary (n = 3, right panel). UNC-104 expression is normalized using β-tubulin as the loading control.
(JPG)

Figure S10 Model for the action of SAM-4 in regulating UNC-104 processivity.
(JPG)

Figure S11 Upstream ORF of *sam-4* message encodes an APC13 homolog. (A) Structure of the F59E12.11 (*sam-4*) transcript from *C. elegans* and other nematodes showing the position of the Anaphase Promoting Complex 13-like (APC13) ORF (blue) and the SAM-4 ORF (black). The 3′, central, and 5′ UTR are shown in grey. cDNAs for the *C. briggsae* and *B. malayi* genes have not been identified, and thus the 3′ and 5′ UTR are in white with grey surround. Six base pairs (bp) separate the ORFs in *elegans* and *briggsae*, and 34 bp separates the ORFS in *malayi*. In all vertebrates examined, the genes encoding *APC13* and *SAM4* (a.k.a. *LOH12CR1*) homologs are not linked. (B) Alignment of *Homo sapiens* APC13 and APC13 homologs the frog *Xenopus tropicalis*, the fly *Drosophila melanogaster*, the soil nematode *C. elegans*, the human pathogenic nematodes *Brugia malayi*, and *Loa loa*, the plant pathogenic nematodes *Globodera rostochiensis* and *Heterodera glycines*, the mycorrhizal fungus *Glomus intraradices* and the plant *Arabidopsis thaliana*. (C) Alignment of the DNA sequences coding for the APC13 like ORF from *C. elegans*, *C. brenneri*, and *C. briggsae* showing substitution patterns. Synonymous substitutions are labeled in red, and, and non-synonymous substitutions are labeled in green.
(PDF)

Table S1 *C. elegans* strains used in this study.
(DOCX)

Table S2 Oligonucleotide primers used in this study.
(DOC)

Movie S1 Movie of SV marker GFP-RAB-3 trafficking (green) along with SAM-4-TagRFP (red).
(AVI)

Movie S2 Movie of SV marker GFP-RAB-3 trafficking.
(AVI)

Movie S3 Movie of SAM-4-TagRFP trafficking.
(AVI)
