# Supplementary figures and images for: The Vesicle Protein SAM-4 Regulates the Processivity of Synaptic Vesicle Transport
Source: PLoS Genet. 2014 Oct 16;10(10):e1004644. doi: 10.1371/journal.pgen.1004644 (PMC4199485; doi:10.1371/journal.pgen.1004644)

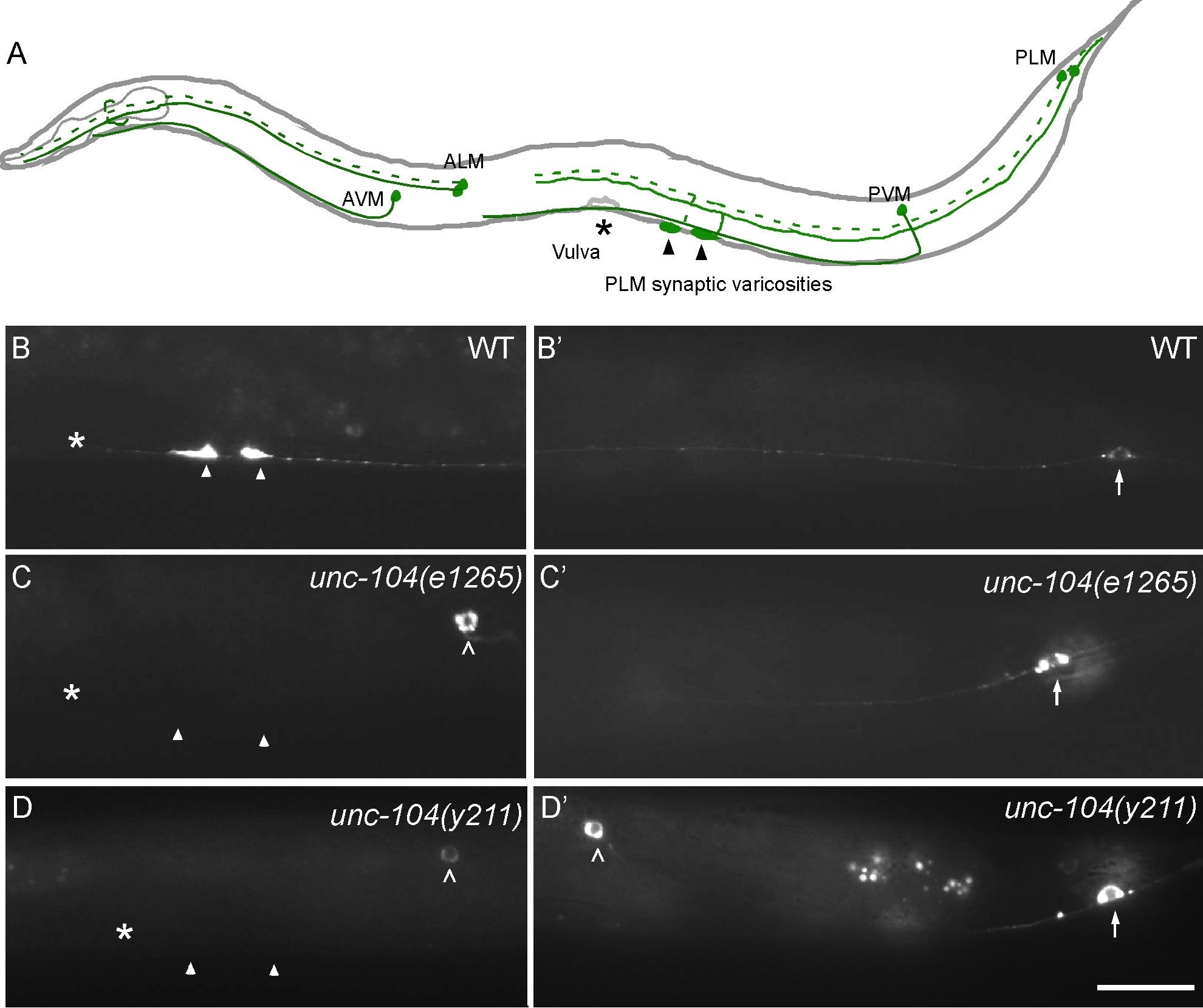

Supplement: Figure S1 — SV trafficking in PLM neurons in unc-104 mutants. (A) Diagram of C. elegans mechanosensory system anatomy. (B–D′) Distribution of GFP-RAB-3 (jsIs821) accumulations in PLM synaptic varicosities (B, C and D) and PLM soma (B′, C′ and D′) in L4 wild type and unc-104 mutants. The D1497N PH domain unc-104(e1265) mutant is homozygous viable but severely uncoordinated. The H215Y motor domain unc-104(y211) mutant is sub-viable. Most animals arrest as L1 larvae, but under optimal growth conditions (moist plates at 15°C), a subset of animals eventually reach adulthood and occasionally produce progeny. However, the strain is difficult to maintain as a homozygous stock. Arrowheads: PLM synaptic varicosities; arrows: PLM soma; carets: PVM soma; scale bar: 20 µm. (JPG) [file pgen.1004644.s001.jpg]

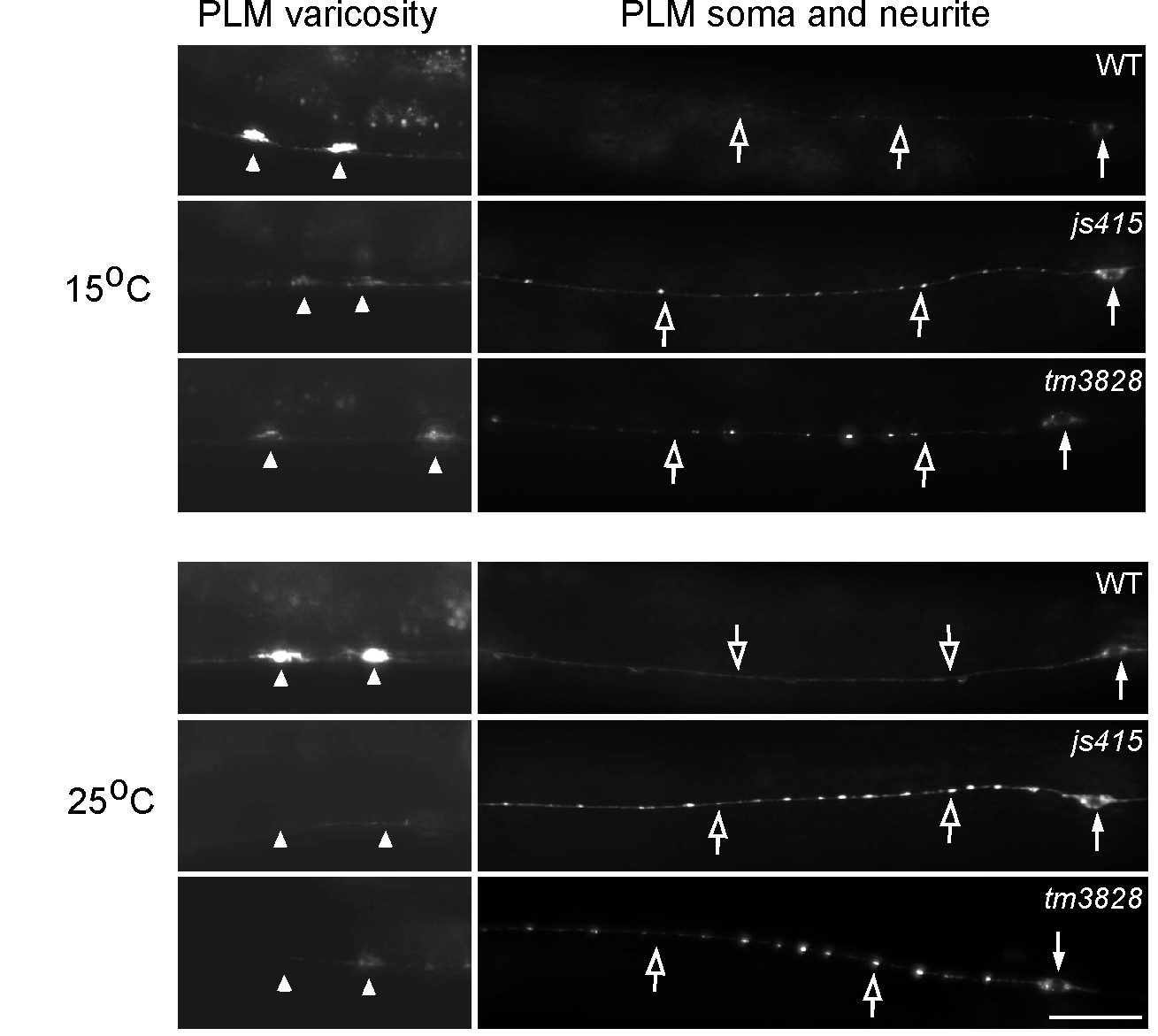

Supplement: Figure S2 — Temperature sensitivity of sam-4(js415) and sam-4(tm3828). Animals were grown at different temperatures as shown and the GFP-RAB-3 signal (jsIs821) in L4 animals was imaged under the same illumination and camera settings. Arrowheads: PLM synaptic varicosities; solid arrows: PLM soma; open arrows: PLM proximal neurites; asterisk: vulva. Scale bar: 20 µm. (JPG) [file pgen.1004644.s002.jpg]

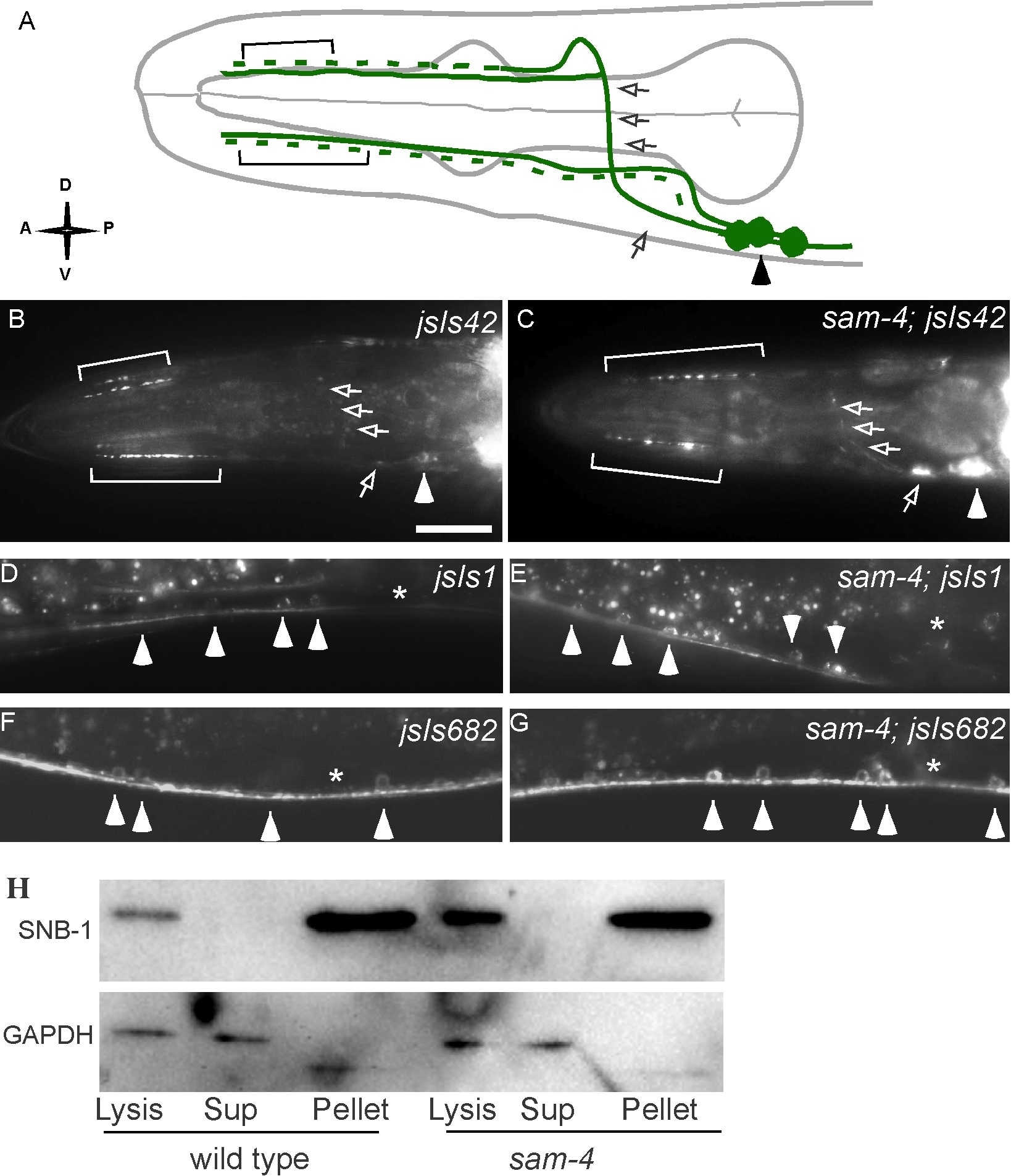

Supplement: Figure S3 — SV distribution in SAB motor neurons. (A) Diagram of the anatomy of C. elegans SAB neurons. (B and C) SNB-1-GFP distribution in SAB neurons of jsIs42 (unc-4p::snb-1-GFP) in wild type (B) and sam-4(js415) (C) animals. Bracket: distal region of the SAB neurites; open arrows: proximal region of SAB neurites; solid arrows: SAB soma. (D and E) SNB-1-GFP distribution in ventral nerve cord neurons of jsIs1 (snb-1p::snb-1-GFP) in wild type (D) and sam-4(js415) (E) animals. (F and G) GFP-RAB-3 distribution in ventral nerve cord neurons of jsIs682 (rab-3p::GFP-rab-3) in wild type (F) and sam-4(js415) (G) animals. Solid arrowheads: neuron soma; asterisk: vulva. Scale bar: 20 µm. (H) Western blot of fractionated worm lysis for SV protein SNB-1 and cytosolic marker GAPDH in wild type and sam-4 animals (See Results for cell fractionation details). (JPG) [file pgen.1004644.s003.jpg]

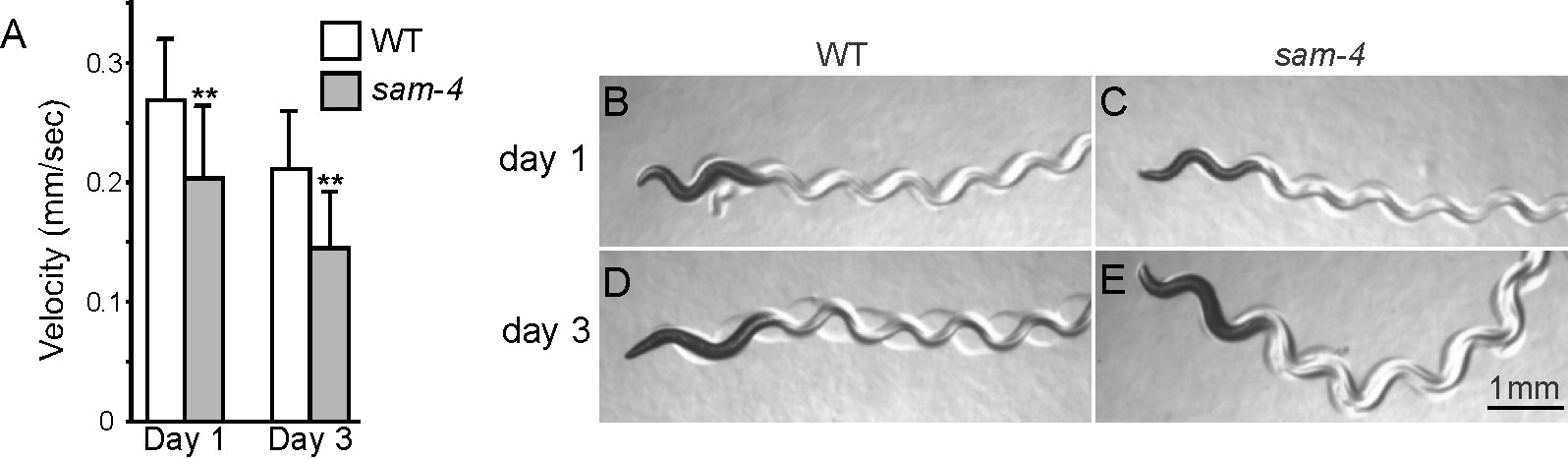

Supplement: Figure S4 — Behavioral defects of sam-4(js415) mutants. (A) Stimulated velocity of moving animals of different ages grown at 25°C. L4 animals were transferred to fresh food for tests at day 0 (**: P<0.001). (B–E) Posture of wild type and sam-4 animals 1 day (B and C) and 3 days (D and E) after molting to the adult stage. (JPG) [file pgen.1004644.s004.jpg]

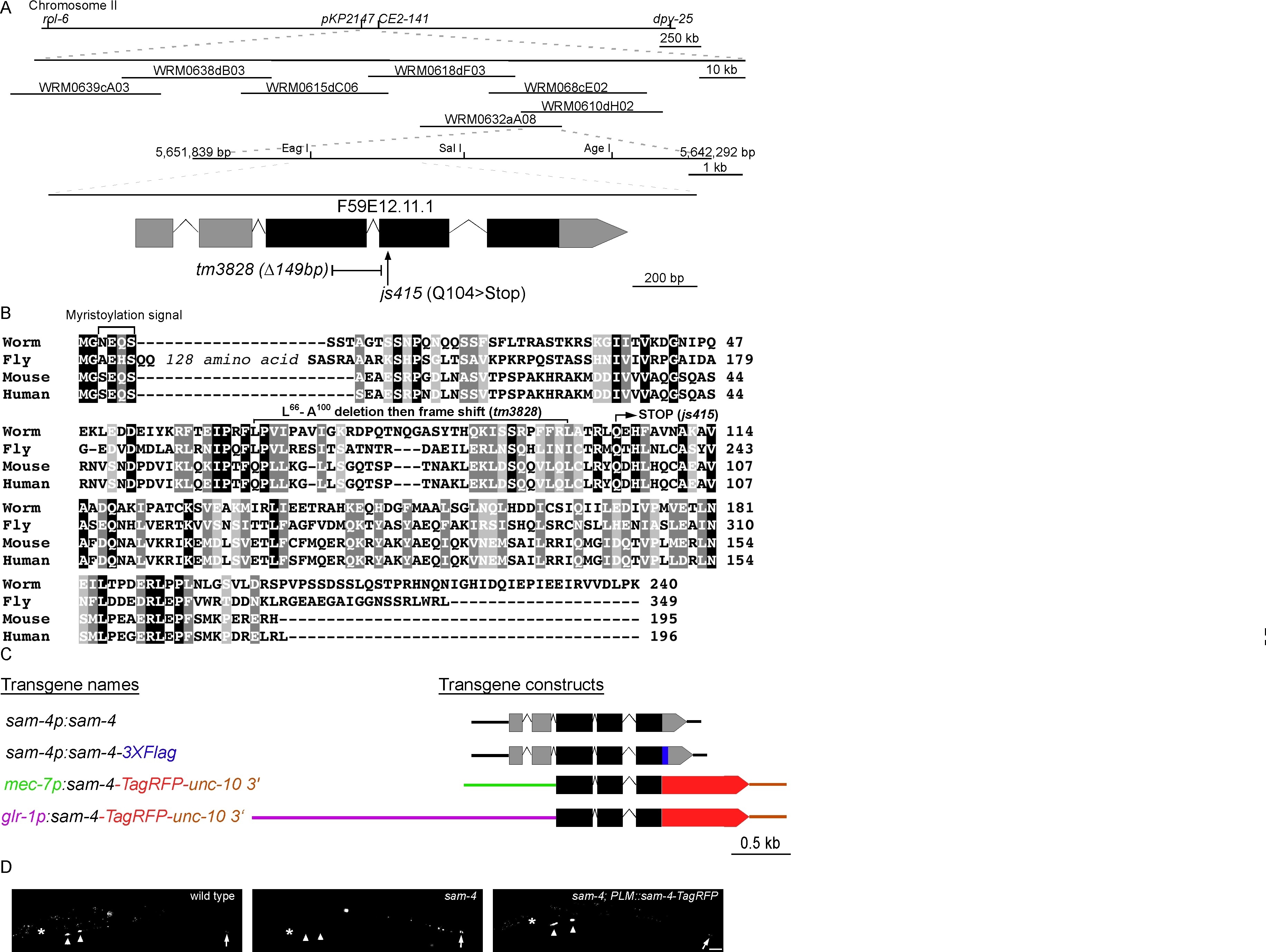

Supplement: Figure S5 — Molecular genetic characterization of the sam-4 gene. (A) Diagram of the genomic organization of the dpy-25- rol-6 region of chromosome II and the structure of the sam-4 gene with genetic lesions shown. The tm3828 junction fragment is caaaatgttcttgtaatcgtgttgΔaaaacggggaatttcggtgaatctt. The js415 is a C>T transition in the following sequence actagcaacacgatta[C/T]aagaacattttgcc. (B) Alignment of SAM-4 with its orthologs in fly, mouse and human. The conserved N-terminal myristoylation consensus sequence (MGXXX[S/T] [48]) and molecular lesions in js415 and tm3828 are indicated. (C) Diagram of sam-4 transgene constructs used in analyzing SAM-4 activity. sam-4 exons are in black and untranslated regions of the message in gray. Distinct promoters, fluorescent proteins, molecular tags, and 3′ UTRs are color coded in the diagram. (D) Defects of SV accumulation in PLM synaptic varicosities in sam-4(js415) mutants were rescued by expression of sam-4 transgene cell autonomously. Arrowheads: PLM synaptic varicosities; arrows: PLM soma; asterisk: vulva; scale bar: 20 µm. (JPG) [file pgen.1004644.s005.jpg]

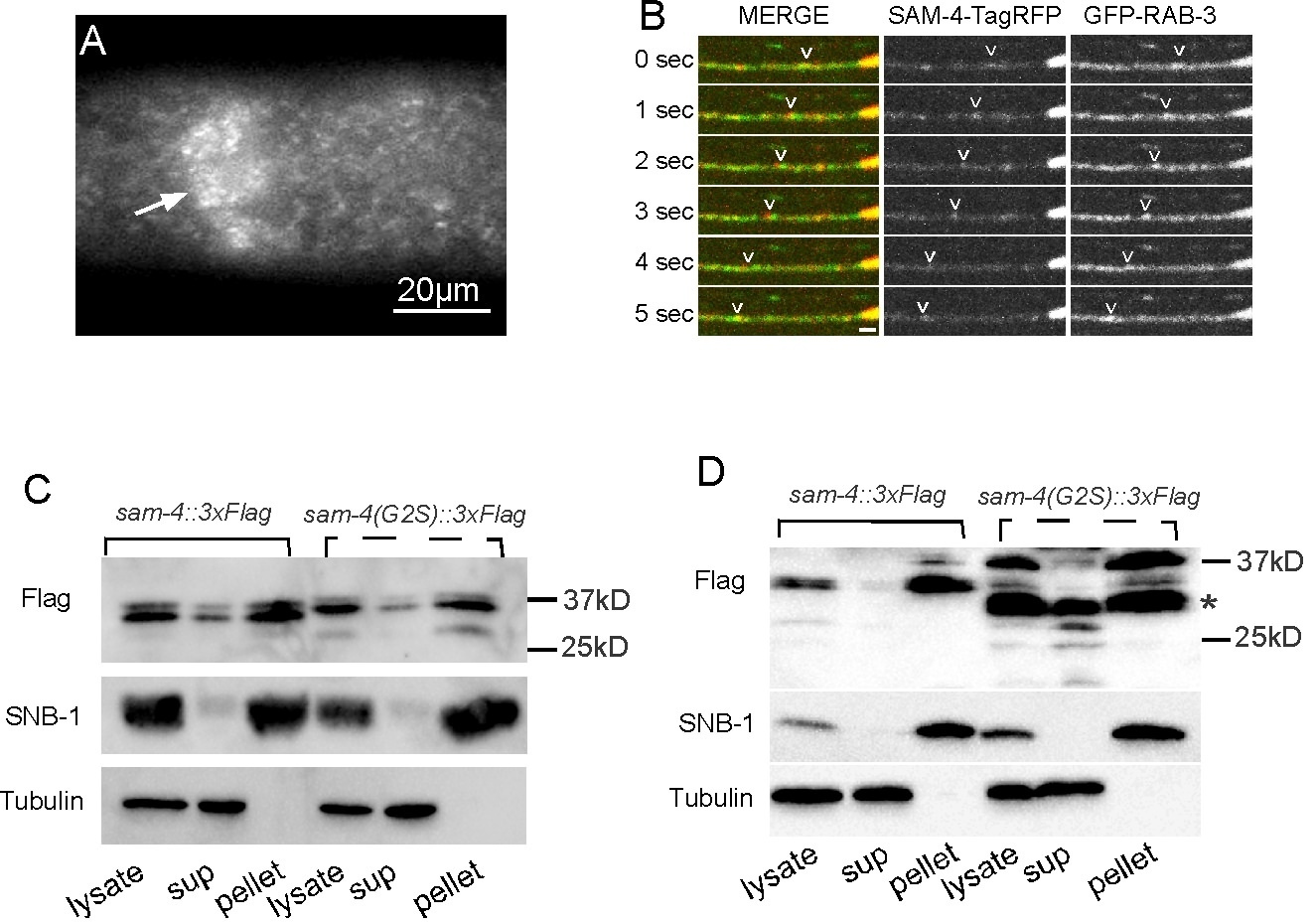

Supplement: Figure S6 — Localization of SAM-4. (A) Anti-FLAG immunohistochemistry of a single copy sam-4-3XFlag transgene (jsIs1188) expressed under the sam-4 promoter in sam-4(js415). Expression was detected in the nerve ring (arrow). Scale bar: 20 µm. (B) Co-localization of moving GFP-RAB-3 (jsIs821) and SAM-4-TagRFP (jsIs1156) particles in a PLM neurite. Recorded fragment is 30 µm away from the PLM soma. Anterograde is to the left. Note that the SAM-4-TagRFP particle is just ahead of GFP-RAB-3 particle due to time lapse during acquiring fluorescent signal alternatively: at each time point, the GFP signal was captured first for 0.33 sec followed by RFP for 0.33 sec. Scale bar: 1 µm. (C–D) Western blots of cell fractions of different genotypes as indicated with EGTA EDTA containing (C) and with non-EGTA/EDTA containing (D) buffer. (JPG) [file pgen.1004644.s006.jpg]

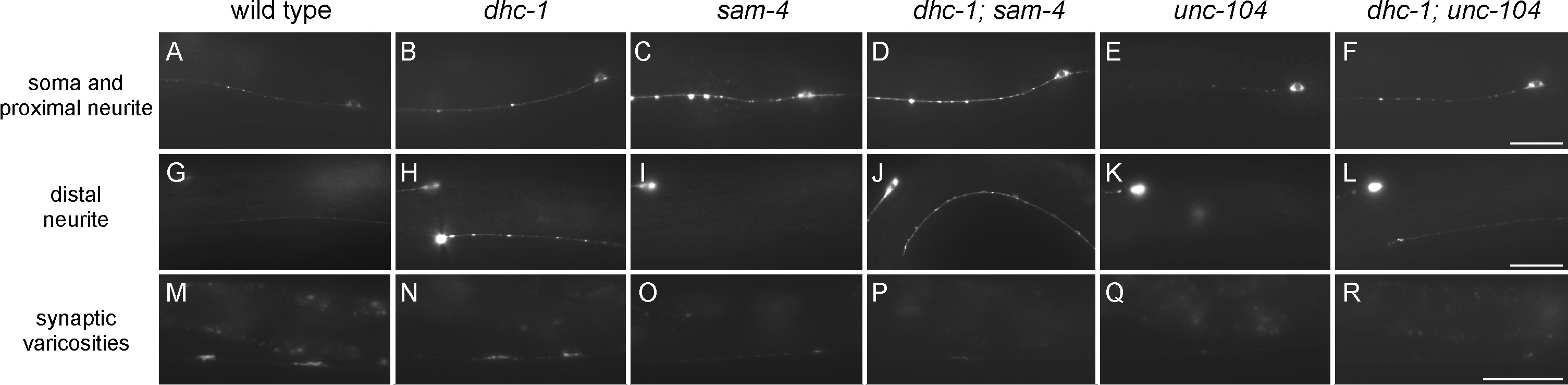

Supplement: Figure S7 — Genetic interactions of sam-4 and unc-104 with dhc-1. Distribution of GFP-RAB-3 accumulations in the PLM soma (A–F), the distal (most anterior) portion the PLM neurite (G–L) and the PLM synaptic varicosities (M–R) in different genetic backgrounds as indicated. Alleles tested: sam-4(js415), dhc-1(js319) and unc-104(js901). Scale bar: 20 µm. (JPG) [file pgen.1004644.s007.jpg]

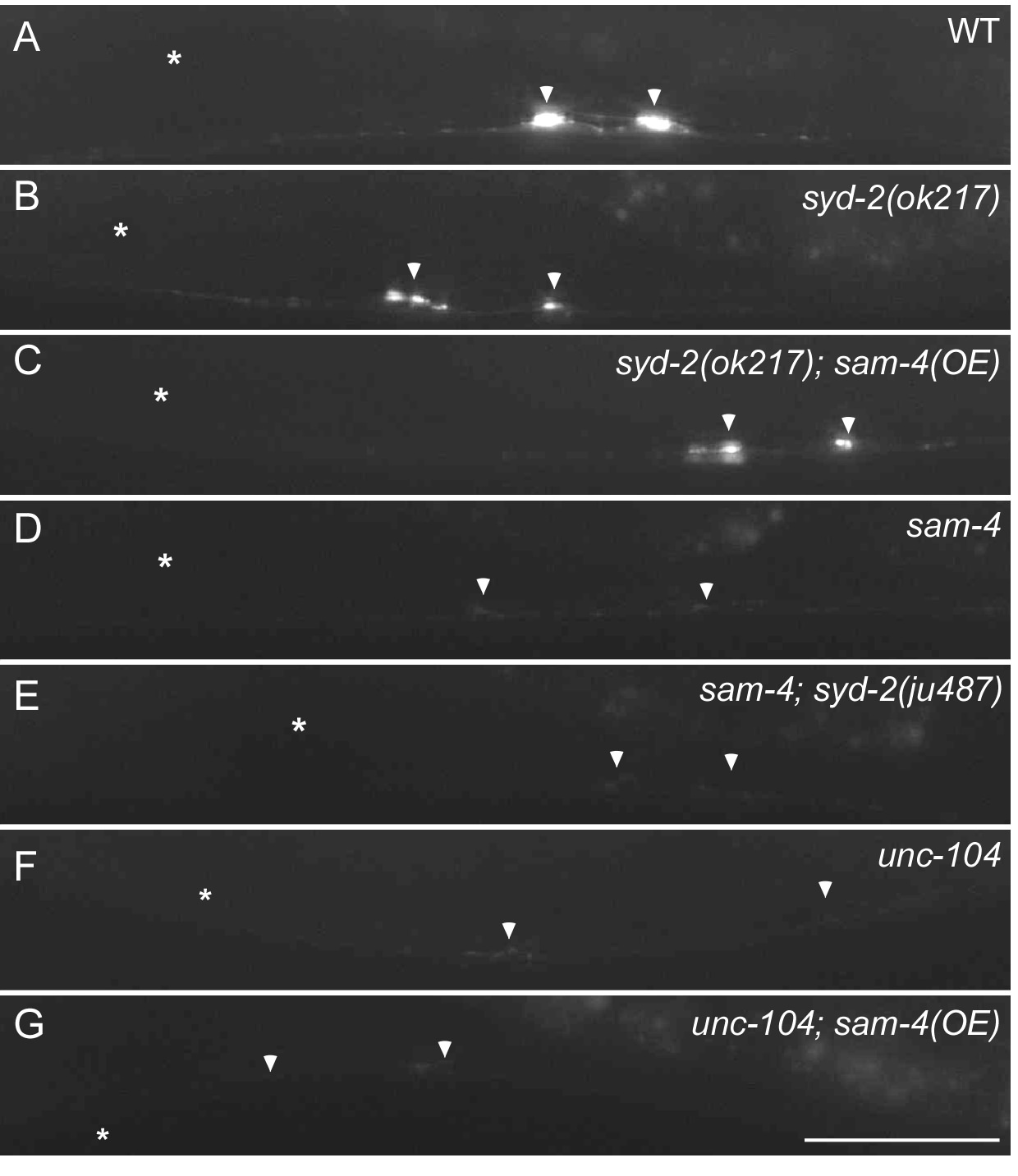

Supplement: Figure S8 — Gain-of-function genetic tests on sam-4(js415), syd-2(ok217) and unc-104(js901) mutations. A–G) Shown is the distribution of GFP-RAB-3 accumulations in PLM synaptic varicosities in different genetic backgrounds. sam-4 over expression (sam-4(OE)) was examined using a sam-4 transgene (jsIs1156) expressed in PLM neurons, while syd-2(ju487) was used for syd-2 gain-of-function tests. Alleles used: sam-4(js415) and unc-104(js901).Arrowheads: PLM synaptic varicosities; asterisk: vulva. Scale bar: 20 µm. (JPG) [file pgen.1004644.s008.jpg]

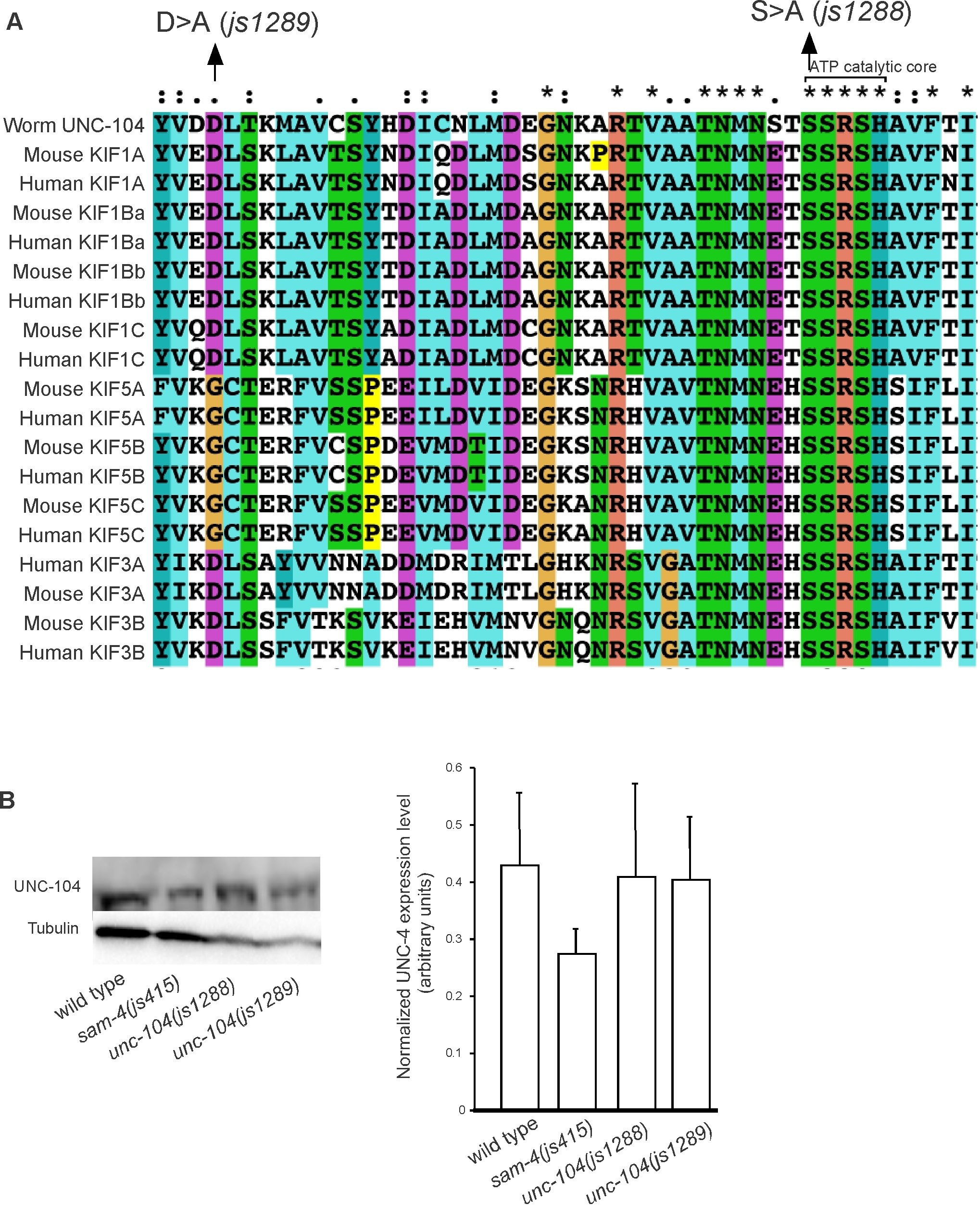

Supplement: Figure S9 — Characterization of UNC-104 motor domain mutants. (A) KIF1A/UNC-104 alignment focusing on the motor region containing the mutated residues in our isolated unc-104 alleles and the ATP catalytic core as indicated. (B) Western blot for UNC-104 expression levels in different genetic backgrounds (left panel) and its quantification summary (n = 3, right panel). UNC-104 expression is normalized using β-tubulin as the loading control. (JPG) [file pgen.1004644.s009.jpg]

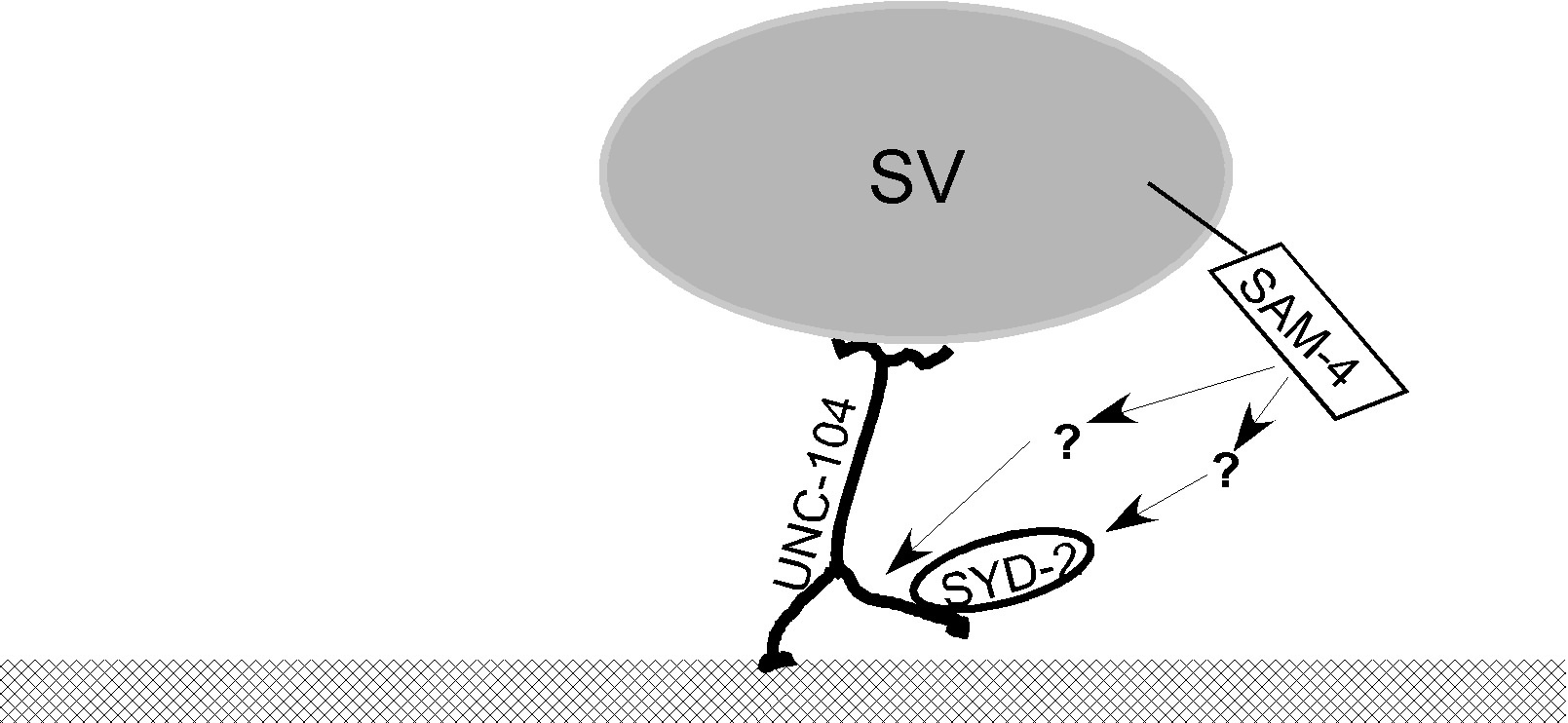

Supplement: Figure S10 — Model for the action of SAM-4 in regulating UNC-104 processivity. (JPG) [file pgen.1004644.s010.jpg]
