# Supplementary material for: The Vesicle Protein SAM-4 Regulates the Processivity of Synaptic Vesicle Transport
Source: PLoS Genet. 2014 Oct 16;10(10):e1004644. doi: 10.1371/journal.pgen.1004644 (PMC4199485; doi:10.1371/journal.pgen.1004644)
Supplement: Figure S11 — Upstream ORF of sam-4 message encodes an APC13 homolog. (A) Structure of the F59E12.11 (sam-4) transcript from C. elegans and other nematodes showing the position of the Anaphase Promoting Complex 13-like (APC13) ORF (blue) and the SAM-4 ORF (black). The 3′, central, and 5′ UTR are shown in grey. cDNAs for the C. briggsae and B. malayi genes have not been identified, and thus the 3′ and 5′ UTR are in white with grey surround. Six base pairs (bp) separate the ORFs in elegans and briggsae, and 34 bp separates the ORFS in malayi. In all vertebrates examined, the genes encoding APC13 and SAM4 (a.k.a. LOH12CR1) homologs are not linked. (B) Alignment of Homo sapiens APC13 and APC13 homologs the frog Xenopus tropicalis, the fly Drosophila melanogaster, the soil nematode C. elegans, the human pathogenic nematodes Brugia malayi, and Loa loa, the plant pathogenic nematodes Globodera rostochiensis and Heterodera glycines, the mycorrhizal fungus Glomus intraradices and the plant Arabidopsis thaliana. (C) Alignment of the DNA sequences coding for the APC13 like ORF from C. elegans, C. brenneri, and C. briggsae showing substitution patterns. Synonymous substitutions are labeled in red, and, and non-synonymous substitutions are labeled in green. (PDF) [file pgen.1004644.s011.pdf]

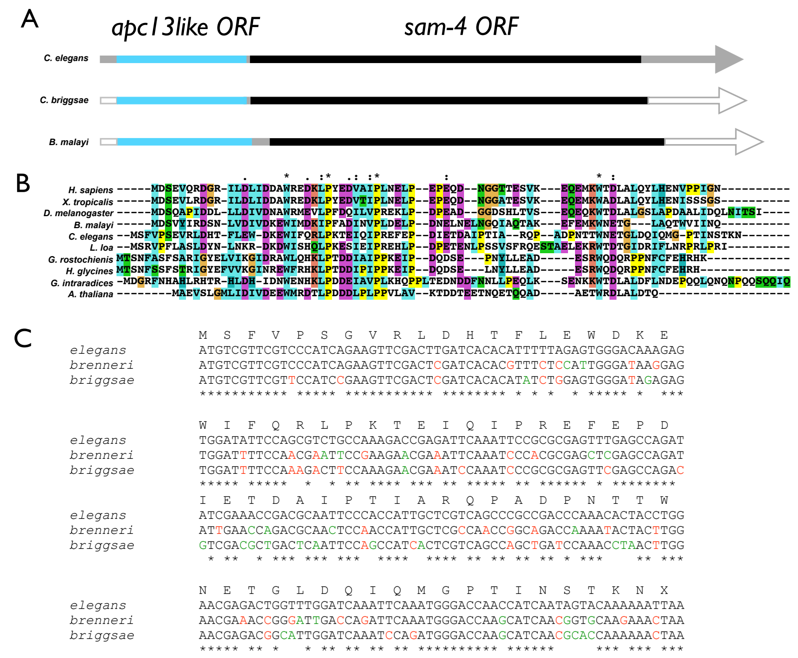
A
apc13like ORF
sam-4 ORF
C. elegans
C. briggsae
B. malayi
B
H. sapiens
X. tropicalis
D. melanogaster
B. malayi
C. elegans
L. loa
G. rostochienis
H. glycines
G. intraradices
A. thaliana
C
M S F V P S G V R L D H T F L E W D K E
elegans ATGTCGTTCGTCCCATCAGAAGTTCGACTTGATCACACATTTTTAGAGTGGGACAAAGAG
brenneri ATGTCGTTCGTCCCATCAGAAGTTCGACTCGATCACACGTTTCTCCATTGGGATAAGGAG
briggsae ATGTCGTTCGTTCCATCCGAAGTTCGACTCGATCACACATATCTGGAGTGGGATAGAGAG
W I F Q R L P K T E I Q I P R E F E P D
elegans TGGATATTCCAGCGTCTGCCAAAGACCGAGATTCAAATTCCGCGCGAGTTTGAGCCAGAT
brenneri TGGATTTTCCAACGAATTCCGAAGAACGAAATTCAAATCCCACGCGAGCTCGAGCCAGAT
briggsae TGGATTTTCCAAAGACTTCCAAAGAACGAAATCCAAATCCCGCGCGAGTTCGAGCCAGAC
I E T D A I P T I A R Q P A D P N T T W
elegans ATCGAAACCGACGCAATTCCCACCATTGCTCGTCAGCCCGCCGACCCAAACACTACCTGG
brenneri ATTGAACCAGACGCAACTCCAACCATTGCTCGCCAACCGGCAGACCAAAATACTACTTGG
briggsae GTCGACGCTGACTCAATTCCAGCCATCACTCGTCAGCCAGCTGATCCAAACCTAACTTGG
N E T G L D Q I Q M G P T I N S T K N X
elegans AACGAGACTGGTTTGGATCAAATTCAAATGGGACCAACCATCAATAGTACAAAAAATTAA
brenneri AACGAAACCGGGATTGACCAGATTCAAATGGGACCAAGCATCAACGGTGCAAGAAACTAA
briggsae AACGAGACGGCATTGGATCAAATCCAGATGGGACCAAGCATCAACGCACCAAAAAACTAA
