# Supplementary material for: The Vesicle Protein SAM-4 Regulates the Processivity of Synaptic Vesicle Transport
Source: PLoS Genet. 2014 Oct 16;10(10):e1004644. doi: 10.1371/journal.pgen.1004644 (PMC4199485; doi:10.1371/journal.pgen.1004644)
Supplement: Table S1 — C. elegans strains used in this study. (DOCX) [file pgen.1004644.s012.docx]

Strains used in this study

| **Strain^1^** | **Genotype** | **Reference^2^** |
| --- | --- | --- |
| N2 | Bristol wild type | Brenner 1974 |
| CB4856 | Hawaiian wild type | Hodgkin and Doniach 1997 |
| NM3861 | *sam-4(js415) II* | This study |
| NM3873 | *sam-4(tm3828) II* | This study |
| NM2689 | *jsIs821* [*mec-7_p_:GFP-rab-3, CBunc119] X* | Bounoutas et al. 2009 |
| NM2879 | *sam-4(js415) II; jsIs821 X* | This study |
| NM3876 | *sam-4(tm3828) II; jsIs821 X* | This study |
| NM306 | *jsIs1* [*snb-1_p_::SNB-1-GFP, pRF4 (rol-6)*] I | Nonet 1999 |
| NM4603 | *sam-4(js415) II; jsIs1* | This study |
| NM2396 | *jsIs682* [*rab-3_p_::GFP-rab-3 lin-15) III* | Nonet 1999 |
| NM4602 | *sam-4(js415) II; jsIs682* | This study |
| NM3336 | *jsIs973* [*mec-7_p_:mRFP, CBunc119*] *III* | Zheng et al. 2011 |
| NM3586 | *sam-4(js415) II; jsIs973 III; jsIs821 X* | This study |
| NM3573 | *jsIs1073* [*mec-7_p_::TagRFP-mito* *,CBunc119*] | This study |
| NM3715 | *sam-4(js415) II; jsIs1073; jsIs821 X* | This study |
| NM3574 | *jsIs1075* [*mec-7_p_::TagRFP-elks-1 CBunc119*] | Zheng et al. 2011 |
| NM3689 | *sam-4(js415) II; jsIs1075; jsIs821 X* | This study |
| NM3869 | *unc-119(ed3) III; jsIs1156 [mec-7_p_::sam-4-TagRFP, CBunc119] IV* | This study |
| NM3863 | *sam-4(js415) II; jsIs1156 IV; jsIs821 X* | This study |
| NM3933 | *jsIs1188 [sam-4_p_-sam-4-3XFlag, CB unc-119] IV* | This study |
| NM4153 | *sam-4(js415) II; jsIs1188 IV* | This study |
| NM3967 | *sam-4(js415) II; jsIs1188 IV; jsIs821 X* | This study |
| NM4211 | *sam-4(js415) II; jsIs1265 [sam-4_p_-sam-4(G2S)-3XFlag, CBunc119] IV* | This study |
| NM4661 | *sam-4(js415) II; jsIs821; jsEx1340[mec-7p::sam-4-TagRFP; myo-2p::GFP]* | This Study |
| NM3934 | *jsIs1189 [glr-1_p_::sam-4-TagRFP, CBunc119] IV* | This study |
| NM3964 | *sam-4(js415)II; jsIs1189 IV; jsIs821 X* | This study |
| NM3863 | *sam-4(js415) II; jsIs1156 IV; jsIs821 X* | This study |
| NM4467 | *unc-104(js1288) II; jsIs821 X* | This study |
| NM4461 | *unc-104(js1289) II; jsIs821 X* | This study |
| NM4368 | *sam-4(js415) unc-104(js1288) II; jsIs821 X* | This study |
| NM4388 | *sam-4(js415) unc-104(js1289) II; jsIs821 X* | This study |
|  | *unc-104(js1288) II; syd-2(ok217) jsIs821 X* | This study |
| NM4548 | *unc-104(js1289) II; syd-2(ok217) jsIs821 X* | This study |
| NM654 | *jsIs42 [unc-4_p_::snb-1-GFP; pJM23[lin-15]] X* | Nonet 1999 |
| NM2081 | *sam-4(js415) II; jsIs42 X* | This study |
| NM4551 | *unc-104(y211) rol-6(e187)/mC6g II; jsIs821 X* | Kumar et al. 2010 |
| NM3071 | *unc-104(js901) jsIs821 II* | This study |
| NM3106 | *rab-3(js49) unc-104(js901) II; jsIs821 X* | This study |
| NM3886 | *+ bli-2(e768) unc-104(js901) / sam-4(js415) + unc-104(js901) II; jsIs821 X* | This study |
| NM354 | *unc-104(e1265) II* | Hall et al. 1991 |
| NM4550 | *unc-104(e1265) II; jsIs821 X* | This study |
| NM4258 | *+ bli-2(e768) + rol-6(e187) / sam-4(js415) + unc-104(e1265) + II; jsIs821 X* | This study |
| NM1442 | *unc-104(rh43) II* | Hall et al. 1991 |
| NM4253 | *+ bli-2(e768) + rol-6(e187) / sam-4(js415) + unc-104(rh43) + II* | This study |
| NM2772 | *syd-2(ok217) X* | Wagner et al. 2009 |
| NM3031 | *syd-2(ju487) jsIs821 X* | This study |
| NM4251 | *sam-4(js415) II; syd-2(ju487) jsIs821 X* | This study |
| NM4279 | *sam-4(js415) II; syd-2(ok217) jsIs821 X* | This study |
| NM4250 | *syd-2(ok217) jsIs821 X* | This study |
| NM4270 | *sam-4(js415) II; syd-2(ok217) jsIs821 X* | This study |
| NM4189 | *jsIs1263 [mec-7p::TagRFP-rab-3-RIM3’ cbunc-119(+)] II; unc-119(ed3) III* | This study |
| NM3764 | *jsIs1111 [mec-4p::unc-104-GFP cbunc-119(+)]* | Kumar et al. 2010 |
| NM4489 | *sam-4(js415) jsIs1263 II; jsIs1111* | This study |
| NM4181 | *dhc-1(js319) I; jsIs821 X* | Murthy et al. 2011 |
|  | *dhc-1(js319) I; sam-4(js415) II; jsIs821 X* | This study |
| NM4549 | *dhc-1(js319) I; unc-104(js901) II; jsIs821 X* | This study |
| NM4497 | *jsIs1263 II; jsIs1111* | This study |
|  | *unc-104(js901) II; syd-2(ok217) jsIs821 X* | This study |
| *NM4487* | *unc-104(e1265) II; syd-2(ok217) jsIs821 X* | This study |
|  | *unc-104(rh43) II; syd-2(ok217) X* | This study |
|  | *unc-104(js901) II; syd-2(ok217) X* | This study |

^1^ Strains without strain numbers are not available as frozen homozygous stocks either from our lab or the CGC. In most cases this is because the stock is not maintainable as a homozygote because of poor growth and/or fertility. In a few cases, the animals were analyzed but not frozen, but in these cases the stocks can easily be rebuilt by simple crosses.

^2^References

**Bounoutas, A., Zheng, Q., Nonet, M. L. and Chalfie, M.** (2009). mec-15 Encodes an F-box Protein Required for Touch Receptor Neuron Mechanosensation, Synapse Formation, and Development. *Genetics* **183**, 607-617.

**Brenner, S.** (1974). The genetics of *Caenorhabditis elegans. Genetics* **77**, 71-94.

**Hall, D. H. and Hedgecock, E. M.** (1991). Kinesin-related gene *unc-104* is required for axonal transport of synaptic vesicles in *C. elegans. Cell* **65**, 837-847.

**Hodgkin, J. and Doniach, T.** (1997). Natural variation and copulatory plug formation in Caenorhabditis elegans. *Genetics* **146**, 149-164.

**Kumar, J., Choudhary, B. C., Metpally, R., Zheng, Q., Nonet, M. L., Ramanathan, S., Klopfenstein, D. R. and Koushika, S. P.** (2010). The Caenorhabditis elegans Kinesin-3 motor UNC-104/KIF1A is degraded upon loss of specific binding to cargo. *PLoS Genet* **6**, e1001200.

**Murthy, K., Bhat, J. M. and Koushika, S. P.** (2011). In vivo imaging of retrogradely transported synaptic vesicle proteins in Caenorhabditis elegans neurons. *Traffic* **12**, 89-101.

**Nonet, M. L.** (1999). Visualization of synaptic specializations in live *C. elegans* using synaptic vesicle-GFP protein fusions. *J. Neurosci. Methods* **89**, 33-40.

**Wagner, O. I., Esposito, A., Kohler, B., Chen, C. W., Shen, C. P., Wu, G. H., Butkevich, E., Mandalapu, S., Wenzel, D., et al.** (2009). Synaptic scaffolding protein SYD-2 clusters and activates kinesin-3 UNC-104 in C. elegans. *Proc Natl Acad Sci U S A* **106**, 19605-19610.

**Zheng, Q., Schaefer, A. M. and Nonet, M. L.** (2011). Regulation of C. elegans presynaptic differentiation and neurite branching via a novel signaling pathway initiated by SAM-10. *Development* **138**, 87-96.
