# Supplementary material for: The Vesicle Protein SAM-4 Regulates the Processivity of Synaptic Vesicle Transport
Source: PLoS Genet. 2014 Oct 16;10(10):e1004644. doi: 10.1371/journal.pgen.1004644 (PMC4199485; doi:10.1371/journal.pgen.1004644)
Supplement: Table S2 — Oligonucleotide primers used in this study. (DOC) [file pgen.1004644.s013.doc]

**Table S2 PCR primers used in this study**

| **Primer** | **Primer sequence** |
| --- | --- |
| 3645 | 5’ tgactctaggtacctcatagttgatcaacacttgg |
| 3571 | 5’ cccttggctagcgtcgacggtaccatggtgtctaagggcgaagag |
| 3572 | 5’ atggtgcgggaccaggtgccataccgtacgcattaagtttgtgcc |
| 3646 | 5’ ttggttctctgcagctcgcattctttgctggtgatc |
| 3985 | 5’ tgattataaagatcatgacatcgattacaaggattaattaatttgtaaatttttattctcttc |
| 3986 | 5’ tgatctttataatcaccgtcatggtctttgtagtcttttggtagatcaactacgc |
| 4059 | 5’ acaaattttcagatgtcaaacgaacaaagctc |
| 4060 | 5’ gagctttgttcgtttgacatctgaaaatttgt |
| 3702 | 5’ aaaagctagcatgggaaacgaacaaagctcgt |
| 3701 | 5’ tttccatggcttttggtagatcaactacgcgg |
| 3981 | 5’ ttggttctgctagctgtgaatgtgtcagattggg |
| 3984 | 5’ tgactctagcatgcgacaaacctcagaaaccaacc |
